# Cost-Effectiveness of Interventions to Improve Maternal, Newborn and Child Health Outcomes: A WHO-CHOICE Analysis for Eastern Sub-Saharan Africa and South-East Asia

**DOI:** 10.34172/ijhpm.2021.07

**Published:** 2021-03-17

**Authors:** Karin Stenberg, Rory Watts, Melanie Y. Bertram, Kaia Engesveen, Blerta Maliqi, Lale Say, Raymond Hutubessy

**Affiliations:** ^1^Department of Health Systems Governance and Financing, World Health Organization (WHO), Geneva, Switzerland.; ^2^School of Population and Global Health, The University of Western Australia, Crawley, WA, Australia.; ^3^Department of Nutrition for Health and Development, World Health Organization (WHO), Geneva, Switzerland.; ^4^Department of Maternal, Newborn, Child and Adolescent Health and Ageing, World Health Organization (WHO), Geneva, Switzerland.; ^5^Department of Sexual and Reproductive Health and Research, World Health Organization (WHO), Geneva, Switzerland.; ^6^Department of Immunization, Vaccines and Biologicals (IVB), World Health Organization (WHO), Geneva, Switzerland.

**Keywords:** Cost-Effectiveness, Maternal Health, Child health, Sub-Saharan Africa, South-East Asia

## Abstract

**Background:** Information on cost-effectiveness allows policy-makers to evaluate if they are using currently available resources effectively and efficiently. Our objective is to examine the cost-effectiveness of health interventions to improve maternal, newborn and child health (MNCH) outcomes, to provide global evidence relative to the context of two geographic regions.

**Methods:** We consider interventions across the life course from adolescence to pregnancy and for children up to 5 years old. Interventions included are those that fall within the areas of immunization, child healthcare, nutrition, reproductive health, and maternal/newborn health, and for which it is possible to model impact on MNCH mortality outcomes using the Lives Saved Tool (LiST). Generalized cost-effectiveness analysis (GCEA) was used to derive average cost-effectiveness ratios (ACERs) for individual interventions and combinations (packages). Costs were assessed from the health system perspective and reported in international dollars. Health outcomes were estimated and reported as the gain in healthy life years (HLYs) due to the specific intervention or combination. The model was run for 2 regions: Eastern sub-Saharan Africa (SSA-E) and South-East Asia (SEA).

**Results:** The World Health Organization (WHO) recommended interventions to improve MNCH are generally considered cost-effective, with the majority of interventions demonstrating ACERs below I$100/HLY saved in the chosen settings (low-and middle-income countries [LMICs]). Best performing interventions are consistent across the two regions, and include family planning, neonatal resuscitation, management of pneumonia and neonatal infection, vitamin A supplementation, and measles vaccine. ACERs below I$100 can be found across all delivery platforms, from community to hospital level. The combination of interventions into packages (such as antenatal care) produces favorable ACERs.

**Conclusion:** Within each region there are interventions which represent very good value for money. There are opportunities to gear investments towards high-impact interventions and packages for MNCH outcomes. Cost-effectiveness tools can be used at national level to inform investment cases and overall priority setting processes.

## Introduction

In 2017 an estimated 295000 women died from pregnancy or childbirth-related complications, and 5.3 million children under 5 years of age died in 2018.^1^ Deaths are inequitably distributed across the globe – More than half (3.3 million) of all these deaths happened in sub-Saharan Africa (SSA) followed by Central and Southern Asia with 28% (1.8 million).^1^

Most of these deaths are preventable and can be avoided with the right investments. Following the adoption of the Millennium Development Goals (MDGs) in 2000, significant progress was made on goal 4 to reduce child mortality by two thirds, and goal 5 to reduce maternal mortality by three quarters. Recent reports indicate that maternal deaths decreased by 35% between 2000 and 2017 and deaths of children under-five dropped by 59% between 1990 and 2018 (1). Progress was also made on MDG 1 for nutrition: between 1990 and 2015, the global prevalence of underweight among children aged less than 5 declined from 25% to 14%, nearly reaching the target of a 50% reduction.^2^

These achievements represented significant improvements in population health, and were supported by increased coordinated funding from the development community. However, the goals were not universally achieved, and momentum needs to be maintained in order to address the unfinished agenda. The Sustainable Development Goals (SDGs) have set global targets for further reductions in maternal and child mortality, as well as retaining goals on ensuring universal access to sexual and reproductive healthcare.^3^ Within this agenda, good nutrition plays a key role: maternal and child undernutrition is estimated to contribute to 45 percent of deaths in children under five,^4^ and dietary iron deficiency is the fifth leading cause of disability adjusted life years among women of reproductive age.^5^

There are many high-impact interventions to improve maternal, newborn and child health (MNCH) outcomes, for which evidence on effectiveness is well-known.^6^ Still, service uptake remains low across many settings.^7^ Many countries need to invest more and invest smarter. Evidence on cost-effectiveness allows policy-makers to evaluate if they are using currently available resources effectively and efficiently, and how they can best invest to achieve health targets and universal health coverage with limited resources. Whilst MNCH is generally proclaimed a priority area for investment across settings, actual budget allocation may not be sufficient to meet national targets. There is an increasing call for low- and middle-income countries (LMICs) to provide “investment cases” to indicate the value for money of proposed investments, for example in the area of non-communicable diseases.^8^ This applies equally to MNCH, for which the multi-partner Global Financing Facility (GFF) supports the development of investment cases in low-income settings. The GFF country investment cases aim to identify priority interventions to improve the health and nutrition of women, adolescents, and children.^9^ Evidence on locally contextualized cost-effectiveness data can help identify priorities. With a successful strategy, countries can access new financing from the World Bank, and can also be better informed for where to invest existing domestic resources.

As part of the World Health Organization’s (WHO’s) efforts to support Member States in the development of evidence-informed health strategies, estimates on cost-effectiveness of prevention and treatment interventions are generated using standardized methods.^10^ The analysis presented here is part of an update of the WHO-Choosing Interventions that are Cost-Effective (CHOICE) programme of global level work. In addition to the production of global level estimates, the CHOICE platform provides country contextualization tools to enable decision-makers to undertake their own analyses.

The CHOICE approach to cost-effectiveness is unique in three ways. Firstly, generalized cost-effectiveness is used. This is different to incremental cost-effectiveness which considers the value of adding new interventions at the margin of the existing package. The generalized cost-effectiveness analysis (GCEA) approach on the other hand, allows analysts to compare interventions compared to a “null” scenario, without considering the historical investments made. This allows the analysis to also take a critical view of the current package of available interventions, which may not always present the greatest value for money (for more details on the GCEA approach see methods paper in this series).^10^ Secondly, a broad set of currently recommended interventions with adequate evidence are included in the analysis, initially individually and then as packages of care. The analysis applies a common methodology and assumptions across different disease areas, enabling interventions for different diseases to be compared fairly. Thus, here we analyze interventions to improve MNCH outcomes whereas other papers consider other intervention areas,^11,12^ and a separate summary paper considers the overall implications when a range of interventions are combined.^13^ Thirdly, a user-friendly tool kit is available for analysts to input local data and assumptions, to generate their own estimates.

The previous round of WHO-CHOICE cost-effectiveness analysis for MNCH was published in 2005.^14,15^ Among the highly cost-effective interventions identified were antenatal care for pregnant women, breastfeeding support, community-based newborn care, and micronutrient supplementation for children.

The current study represents the first thorough re-analysis of the cost-effectiveness of interventions targeting MNCH outcomes by WHO since this time. The CHOICE methods and analysis platform have been updated and new health impact models developed. WHO Practice Guidelines have been updated in several areas (eg, antenatal care,^16^ intrapartum care, care for small and sick newborn, etc^17^). A broader set of interventions is considered in the new analysis, including nutritional supplementation before and during pregnancy; and an expanded set of vaccines. Furthermore, a user-friendly country contextualization tool has been developed, to accompany the global level analyses.

## Key Messages

**Implications for policy makers**

- Policy-makers in most countries consider cost-effectiveness to be an important criterion when making decisions around what health services to provide. The literature on cost-effectiveness of interventions to improve MNCH outcomes is rich and growing. There are many known high-impact interventions that have been recommended for a long time. However, service coverage remains limited for many interventions and the evidence base needs to be restated to support the case for investment.
- This paper provides an updated set of cost-effectiveness data for interventions that address MNCH outcomes for 2 geographic regions, following the methods of the WHO-CHOICE approach. These estimates provide a reference point for policy-makers to guide discussions around what interventions to include in national service packages to advance universal health coverage and attain the SDGs.
- Service packages will differ across settings based upon epidemiological profile, health budgets and local values. These global models form a starting point for the production of country-specific data to guide local discussions.

**Implications for public**

Suboptimal maternal, newborn and child health (MNCH) outcomes remains a major cause of burden of disease across low- and middle-income countries (LMICs). Many highly cost-effective interventions are not included in current benefit packages provided to the population, or have modest uptake among the population, either because of supply barriers (limited system capacity, low prioritization) or demand barriers (financial barriers, low demand). Estimates on cost-effectiveness can contribute to evidence-based discussions around what to provide in benefit packages. This can ultimately lead to greater investments in interventions that improve MNCH, allowing populations in LMICs to benefit from better health outcomes.

## Methods

We examined the costs and impact on health of interventions to improve MNCH outcomes in 2 regions: Eastern sub-Saharan Africa (SSA-E) and South-East Asia (SEA). The regions are consistent with previous published analyses.^14,15^

For a full account of the methods used in this update of the WHO-CHOICE project, we refer to a separate paper that is published as part of this series.^10^ In this paper we describe specific methodology related to updating the analytical work for interventions targeting MNCH outcomes, including brief overviews of the models and the intervention assumptions used. The analysis used epidemiological and cost data for 2010, for the SSA-E and SEA Global Burden of Disease regions. Countries included in these regions are listed in Table 1.

**Table 1:** Countries Included in the Analysis

**SEA**	**SSA-E**
Cambodia	Burundi
Indonesia	Comoros
Laos	Djibouti
Malaysia	Eritrea
Maldives	Ethiopia
Myanmar	Kenya
Philippines	Madagascar
Sri Lanka	Malawi
Thailand	Mauritius
Timor-Leste	Mozambique
Viet Nam	Rwanda
	Somalia
	South Sudan
	Sudan
	Uganda
	Tanzania
	Zambia

Abbreviations: SEA, South East Asia; SSA-E, Eastern sub-Saharan Africa.

###  Interventions

We considered interventions across the life course from adolescence to pregnancy and child birth, and during years 0-4 of the child. Interventions included immunization, child healthcare, nutrition, reproductive health, and maternal/newborn healthcare that impact on mortality outcomes for pregnant women or women who recently delivered and children 0-4 years – including stillbirths. Interventions were included based on WHO recommendations, and for which an impact model existed to facilitate modelling. The analysis was undertaken using the Spectrum suite of impact models, and is therefore limited to interventions included in Spectrum, in particular the Lives Saved Tool (LiST)^18^ and the Family Planning tool (FamPlan).^19^

We evaluated 37 interventions and 12 packages of combined interventions. These included interventions that allow individuals to exercise rights around deciding their family size (access to contraception, safe abortion); interventions which promote healthy practices and behaviors (eg, breastfeeding); interventions which prevent illness (eg, through immunization); and interventions which manage complications and illness (eg, complications arising at birth or infectious disease in childhood). Table 2 lists interventions with a description including the period of implementation/ life course (target population), the health programme, and service delivery platform.

**Table 2:** Intervention Description and Target for Impact

**Number**	**Intervention Name**	**Short Name**	**Intervention Definition**	**Target Population in Need of Intervention**	**Target for Impact**	**Health Programme**	**Service Delivery Platform**
**Single Interventions **							
**1**	Family planning	MNCH_1. FP	Women of reproductive age (15-49 years) in union are provided with counseling and information on different methods for contraception, as well as the commodities required. This includes both traditional and modern methods such as pills and condoms, injectables, IUD, implant, and sterilization, based on country-specific profile of contraceptive methods use.	Women of reproductive age (15-49 years) in union	Maternal mortality	Maternal and newborn	Primary level care
**2**	Folic acid supplementation	MNCH_2. FAS	All women, from the moment they begin trying to conceive until 12 weeks of gestation, should take a folic acid supplement (400 μg folic acid daily).	Pregnant women	Newborn (0-1 month), Stillbirths	Nutrition	Primary level care, including community
**3**	Safe abortion services	MNCH_3. SA	Safe abortion provided to women seeking to terminate pregnancy. Methods include manual or electric vacuum aspiration and medical abortion (mifepristone followed by a prostaglandin).	Women seeking to terminate pregnancy (incidence of abortion)	Maternal mortality	Maternal and newborn	Primary level care
**4**	Post abortion case management	MNCH_4. PAC	Treatment of women experiencing complications after undergoing unsafe abortions. Complications include haemorrhage, sepsis, peritonitis, and trauma to the cervix, vagina, uterus, and abdominal organs.	Women seeking to terminate pregnancy (incidence of abortion)	Maternal mortality	Maternal and newborn	Hospital
**5**	Calcium supplementation in pregnant women for the prevention and management of pre-eclampsia/eclampsia	MNCH_5. CS	In populations with low dietary calcium intake, daily calcium supplementation (1.5 g–2.0 g oral elemental calcium) is recommended for pregnant women to reduce the risk of pre-eclampsia.	Pregnant women	Maternal mortality	Nutrition; Maternal and newborn	Primary level care
**6**	Daily iron and folic acid supplementation in pregnant women	MNCH_6. DIFA	Daily oral iron and folic acid supplementation with 30 mg to 60 mg of elemental iron and 400 µg (0.4 mg) folic acid is recommended for pregnant women to prevent maternal anaemia, puerperal sepsis, low birth weight, and preterm birth.	Pregnant women	Newborn (0-1 month)	Nutrition; Maternal and newborn	Primary level care, including community
**7**	Balanced energy-protein supplementation to pregnant women living in areas with high food insecurity balanced	MNCH_7. BEPS	In undernourished populations, balanced energy and protein dietary supplementation is recommended for pregnant women to reduce the risk of stillbirths and small for gestational age neonates.	Pregnant women living in areas with high food insecurity (based on income per capita)	Newborn (0-1 month)	Nutrition; Maternal and newborn	Primary level care, including community and outreach
**8**	Tetanus toxoid vaccination	MNCH_8. TT	Two injections of tetanus toxoid vaccine.	Pregnant women	Maternal mortality; Newborn (0-1 month)	Immunization; Maternal and newborn	Primary level care
**9**	Intermittent presumptive treatment of malaria in pregnancy	MNCH_9. IPTM	Intermittent presumptive treatment of malaria of all pregnant women living in areas endemic for *Plasmodium falciparum*.	Pregnant women living in areas endemic for *Plasmodium falciparum*	Maternal mortality; stillbirths	Malaria; Maternal and newborn	Primary level care
**10**	Syphilis detection and treatment in pregnancy	MNCH_10_SYP	Screening pregnant women by rapid plasma reagent test and treatment of sero-positive cases with penicillin.	Pregnant women	Newborn (0-1 month); stillbirths	Maternal and newborn	Primary level care
**11**	Hypertensive disease case management in pregnancy	MNCH_11. CMHD	Management of moderate to severe hypertension without proteinuria.	Pregnant women	Maternal mortality	Maternal and newborn	Primary level care
**12**	Management of pre-eclampsia (mild and severe)	MNCH_12. MPE	Management of hypertension and mild pre-eclampsia through outpatient care; management of severe pre-eclampsia through with magnesium sulfate through inpatient care.	Pregnant women	Maternal mortality	Maternal and newborn	Primary level care
**13**	Ectopic pregnancy case management	MNCH_13.ECT	Surgical intervention (laparoscopy or laparotomy) to interrupt an ectopic pregnancy.	Pregnant women with ectopic pregnancy	Maternal mortality	Maternal and newborn	Hospital
**14**	Neonatal resuscitation	MNCH_14. NR	Detection of breathing problems and resuscitation of newborn when required, using bag and mask.	Newborn	Newborn (0-1 month)	Maternal and newborn	Primary level care
**15**	Clean cord care (clean birth practices)	MNCH_15. CCC	Umbilical cord cleansing, with chlorhexidine or other disinfectant.	Newborn	Newborn (0-1 month)	Maternal and newborn	Primary level care
**16**	Antibiotics for preterm premature rupture of membranes	MNCH_16. PPROM	Hospitalization prior to delivery, with administration of oral antibiotics to women with preterm premature rupture of membranes.	Pregnant women	Maternal mortality	Maternal and newborn	Hospital
**17**	Management of eclampsia with magnesium-sulphate	MNCH_17. MEMS	Management of convulsions associated with eclampsia, occurring ante-, intra- or postpartum.	Pregnant women	Maternal mortality	Maternal and newborn	Hospital
**18**	Management of maternal sepsis	MNCH_18. MMS	Management of sepsis symptoms within 42 days of delivery.	Pregnant women	Maternal mortality	Maternal and newborn	Hospital
**19**	Promotion of breastfeeding	MNCH_19. BF	Promotion of early and exclusive breastfeeding through skilled care providers and community health workers.	Newborn	Newborn (0-1 month) and child (1-59 months)	Nutrition; Maternal and newborn	Primary level care, including community
**20**	Home visits for clean postnatal practices	MNCH_20. CPNP	Home visits within 48 hours of delivery to promote clean practices, specifically that the mother washes her hands frequently, the child lives in a clean environment, and no harmful practices are performed.	Newborn	Newborn (0-1 month)	Maternal and newborn	Community level
**21**	Vitamin A supplementation (0-4 years)	MNCH_21. VAS	Vitamin A supplementation for children 6-59 months of age in countries (or sub-national areas in some cases) where vitamin A deficiency is a public health problem.	Children 6-59 months	Child (1-59 months)	Nutrition; Child	Community level
**22**	Promotion of complementary feeding	MNCH_22. CF	Comprehensive counselling for the caregiver of a child (two full sessions) on the importance of continued breastfeeding after 6 months of age along with information on appropriate complementary feeding practices, through skilled care providers and community health workers.	Children 6-11 months	Child (1-59 months)	Nutrition; Child	Primary level care, including community
**23**	DPT vaccine	MNCH_23. DPT	3 doses of DPT vaccine.	Newborn	Child (1-59 months)	Immunization	Primary level care
**24**	Hib vaccine	MNCH_24. HIB	3 doses of HiB vaccine.	Newborn	Child (1-59 months)	Immunization	Primary level care
**25**	Pneumococcal vaccine	MNCH_25. PCV	3 doses of pneumococcal vaccine.	Newborn	Child (1-59 months)	Immunization	Primary level care
**26**	Rotavirus vaccine	MNCH_26. ROTA	3 doses of rotavirus vaccine.	Newborn	Child (1-59 months)	Immunization	Primary level care
**27**	Pentavalent vaccine (DPT + Hep B + Hib)	MNCH_27. PENTA (DPT + HEPB + HIB)	3 doses of pentavalent vaccine (a combination of five vaccines-in-one to prevent diphtheria, tetanus, whooping cough, hepatitis b and haemophilus influenza type b).	Newborn	Child (1-59 months)	Immunization	Primary level care
**28**	Measles vaccine	MNCH_28. MCV	2 doses of measles vaccine.	Newborn	Child (1-59 months)	Immunization	Primary level care
**29**	Kangaroo mother care	MNCH_29. KMC	Inpatient support to KMC, defined as continuous skin-to-skin contact between a mother and her newborn as well as frequent and exclusive breastfeeding.	Newborn	Newborn (0-1 month)	Maternal and newborn	Hospital
**30**	Full supportive care for premature babies	MNCH_30. FSC	Prematurely born neonates receive hospital-based full supportive care, including KMC, feeding support/IV fluids, infection prevention/management, oxygen provision, management of neonatal jaundice, nasal CPAP/IPPV (as required), and surfactant for respiratory distress syndrome.	Newborn	Newborn (0-1 month)	Maternal and newborn	Hospital
**31**	Case management of severe neonatal infection (sepsis/pneumonia) with full supportive care	MNCH_31. CMSNI	Case management of neonates with suspected sepsis/pneumonia treated with hospital-based full supportive care, including oxygen, IV fluids, IV antibiotics, blood transfusion, phototherapy, etc as needed, in addition to KMC.	Newborn	Newborn (0-1 month)	Maternal and newborn	Hospital
**32**	Facility-based management of neonatal infection (sepsis/pneumonia) with injectable (and oral) antibiotics	MNCH_32. CMNI	Treatment of sepsis and infection at first level facility, with 2 days injectable antibiotics followed by oral amoxicillin for 7 days.	Newborn	Newborn (0-1 month)	Child	Primary level care
**33**	Management of diarrhea through oral rehydration solution and zinc	MNCH_33. ORSzinc	Management of mild and moderate diarrhea with ORS and zinc tablets.	Children 0-59 months	Child (1-59 months)	Child	Community level
**34**	Community-based management of pneumonia	MNCH_34. CCM_P	Home visits for diagnosis and treatment of community-based management of pneumonia in children below the age of 5 years, provided by community health workers.	Children 0-59 months	Child (1-59 months)	Child	Community level
**35**	Antibiotics for treatment of dysentery	MNCH_35. DYS	Children with diarrhea presenting with blood in the stool receive a 3 day course of ciprofloxacin and are re-evaluated after 2 days.	Children 0-59 months	Child (1-59 months)	Child	Primary level care
**36**	Facility-based management of pneumonia	MNCH_36. FCM_P	Management of pneumonia with oral antibiotics.	Children 0-59 months	Child (1-59 months)	Child	Primary level care
**37**	Management of children with severe acute malnutrition	MNCH_37. CMSAM	Integrated management of children with severe acute malnutrition (<-3 Z-score) through outpatient care for cases without medical complication (80%), and inpatient care for cases with medical complications and/or infants younger than 6 months (20%).	Children 0-59 months	Child (1-59 months)	Nutrition; Child	Primary level care, including community
**Packages **							
**P1**	Preventing and managing unplanned pregnancy	MNCH_P1. UPP	Family planning counseling integrated into safe abortion and post-abortion care (3 interventions: includes #1, #3, and #4).	Women seeking to plan pregnancy	Maternal mortality	Maternal and newborn	Primary level care; (hospital level for post-abortion care)
**P2**	Comprehensive antenatal care	MNCH_P2. ANC	A package of antenatal care aligned with WHO guidelines and including tetanus toxoid vaccine, iron supplementation, calcium supplementation, balanced energy supplementation, syphilis detection and treatment, hypertensive disorder case management, MgSO_4_ management of pre-eclampsia, and IPTM where relevant (8 interventions: includes #5-12).	Pregnant women	Maternal mortality; Newborn (0-1 month); Stillbirths	Maternal and newborn	Primary level care
**P3**	Skilled assistance for normal delivery	MNCH_P3. SBA	Skilled assistance with facility-based births, not necessarily EmOC level. Components include immediate assessment and stimulation, support during labor and delivery, active management of the third stage of labour, newborn resuscitation, and clean cord care. (5 interventions, of which #14 and #15 listed above as individual interventions).	Pregnant women	Maternal mortality; Newborn (0-1 month); Stillbirths	Maternal and newborn	Primary level care
**P4**	Skilled assistance for normal delivery + family planning	MNCH_P4. SBA + FP	P3+ integrated postpartum family planning advice and contraceptive provision (6 interventions).	Pregnant women	Maternal mortality; Newborn (0-1 month); Stillbirths	Maternal and newborn	Primary level care
**P5**	Skilled delivery + management of complications	MNCH_P5. SBA + comp	Skilled assistance for normal deliveries with quick and efficient referral to quality emergency obstetric care services when complications arise, + induction of labor + full supportive care for newborn infections (12 interventions).	Pregnant women	Maternal mortality; Newborn (0-1 month); Stillbirths	Maternal and newborn	Primary level care + hospital
**P6**	Skilled delivery + management of complications + family planning	MNCH_P6. SBA + comp + FP	P5+ integrated postpartum family planning advice and contraceptive provision (13 interventions of which most are listed above as individual interventions).	Pregnant women	Maternal mortality; Newborn (0-1 month); Stillbirths	Maternal and newborn	Primary level care + hospital
**P7**	Case management of newborn complications at referral level	MNCH_P7. CMNC	Full supportive care for premature babies + Case management of severe neonatal infection (sepsis/pneumonia) with full supportive care (2 interventions: combines #30 and # 31).	Newborns with complications (prematurity, severe infection)	Newborn (0-1 month)	Maternal and newborn	Hospital
**P8**	Community-based newborn and child care	MNCH_P8. CBNCC	Community-based preventive and curative care (breastfeeding promotion, postnatal visits, vitamin A supplementation, management of infections, pneumonia and diarrhea), (5 interventions, listed above as #19-21 + #33 + #34).	Newborns and children 0-59 months	Newborn (0-1 month) and child (1-59 months)	Maternal and newborn; Child	Community
**P9**	Infant and young child feeding	MNCH_P9. IYCF	Breastfeeding promotion + Complementary feeding promotion + Vitamin A supplementation (3 interventions, listed above as #19, #21, #22).	Newborns and children 0-59 months	Child (1-59 months)	Nutrition; Child	Community and primary level care
**P10**	Routine EPI (measles, diphtheria, pertussis, tetanus, and tuberculosis)	MNCH_P10. EPI	BCG, DTP, Hib, and measles immunization (4 interventions).	Newborns and children 0-59 months	Child (1-59 months)	Immunization	Primary level care
**P11**	Routine EPI + additional vaccines	MNCH_P11. EPI+ROTA+PCV	BCG, DTP, Hib, measles, rotavirus and pneumococcal vaccines (6 interventions).	Newborns and children 0-59 months	Newborn (0-1 month) and child (1-59 months)	Immunization	Primary level care
**P12**	Primary level integrated management of the sick child (includes link to the community)	MNCH_P12. IMCI	Management of diarrhea, dysentery, pneumonia, and severe malnutrition (4 interventions – combines #33, with #35, #36, #37).	Children 0-59 months	Child (1-59 months)	Child	Primary level care

Abbreviations: MNCH, maternal, newborn and child health; IUD, intrauterine device; IPPV, intermittent positive-pressure ventilation; CPAP, continuous positive airway pressure; ORS, oral rehydration solution; WHO, World Health Organization; EmOC, emergency obstetric care; SBA, skilled birth assistance; KMC, Kangaroo mother care; BCG, Bacillus Calmette–Guérin; EPI, Expanded Programme on Immunization; Hib, Haemophilus influenza type b; DPT, diptheria, tetanus toxoids and pertussis.

It should be noted that some relevant interventions for maternal and child health, such as HPV vaccine, malaria and HIV/AIDS testing and treatment, were considered as part of analysis for other programmatic areas within the WHO-CHOICE series update and are therefore presented and discussed in other papers belonging to this series.^11,12^ An exception is intermittent preventive treatment in pregnancy for malaria which we consider here as part of the antenatal care package and thus fit for inclusion. In addition to single interventions, we evaluate 12 packages that follow policy-relevant intervention combinations.

Interventions and packages are evaluated at three coverage levels, 50%, 80% and 95%. Coverage targets for family planning cannot follow the same logic as they do not refer to a health need but a need for regulating pregnancy (which would never reach 100%). The model therefore incorporates a calculation factor for contraceptive use. We apply a factor of 0.72, which was derived by studying current contraceptive prevalence rates in the Organisation for Economic Co-operation and Development (OECD) countries, which according to recent data reach around 71%-72% for any method.^20^ Thus, a 50% coverage for family planning is run in the model as 50% x 0.72 = 36%.

###  Health Outcomes

 Health outcomes were assessed using the Spectrum suite of impact models.

The LiST and FamPlan tools have been described in detail elsewhere.^17,18^ The Spectrum platform translates an increase in service coverage into effects on demography and health outcomes (eg, birth spacing, cause-specific mortality, nutritional status).^21^

 For each intervention/package, the model generates information about the number of deaths that would have occurred in a scenario with zero coverage for the interventions(s) of interest: the “null” scenario. This is compared to a “scale-up” scenario where there is instantaneous scale-up from zero coverage in year 1 to the target coverage (50%, 80% or 95%) in year 2, with target coverage then maintained for 100 years. To generate the “null” for maternal and child interventions, the SPECTRUM software cost-effectiveness tool runs the LiST and FamPlan modules accordingly, generating a scenario where coverage is zero for relevant interventions and the burden of disease increases accordingly.

Results are analyzed by country and year. The model accounts for the synergies in effects and causes such that lives saved are not double counted. Deaths averted include maternal, newborn, child (0-4 years) and stillbirths. Deaths averted are converted into healthy life years (HLYs) gained based on age at time of death, average life expectancy for that age bracket, and the average health state valuation for a life saved from age at death until life expectancy. The model is largely restricted to impact measured in terms of mortality changes, however we did include both the “years of life lost” and (average) “years lived with disability” component for the future stream of life saved by the interventions. This allows us to compare the cost-effectiveness ratios in $/HLY gained with those from other disease areas. Disease weights used in the HLY calculations are from the Global Burden of Disease study, 2010.^22^ The HLY estimations are thus based on DALY data, and the distinction between DALYs and HLYs is a distinction in name only, not in nature (we believe that “HLYs gained” is a more intuitive measure for decision-makers than “DALYs” when considering investments).

The analysis presented here is constrained by the evidence included within the LiST model, and is therefore largely restricted to evaluating impact on mortality. For interventions such as family planning that do not directly impact on mortality, the effect was measured in terms of averted maternal mortality resulting from fewer births. While many interventions are known to also reduce morbidity, unfortunately a lack of reliable data has prevented inclusion of such impact estimates within the LIST model, and therefore the benefits of some interventions are underestimated. For effect sizes used within the analysis see Supplementary file 1.

###  Cost Assumptions

 Costing of interventions followed a standardized framework developed for WHO-CHOICE, and includes patient level delivery costs, programme costs and health system (service delivery) costs. Costs are estimated from the perspective of the government as the health system funder. Costs incurred by patients outside of the direct healthcare (eg, fees for transport) are not included in the analysis.

The GCEA analytical perspective assumes there is sufficient health system capacity in place to support the intervention. Quantity assumptions are based on adherence to WHO guidelines for the intervention of interest, and the analysis uses patient level intervention costs from the OneHealth Tool,^23^ with detailed prices for medicines and supplies, and with an additional 13% markup rate applied to medicine and supply prices to cover logistics costs.^24^ Programme costs follow a standard methodology,^24^ with prices from the WHO-CHOICE price database (https://www.who.int/choice) and capital expenses annuitized over the lifetime of the good. Health system (service delivery) costs use WHO-CHOICE country-specific estimates for inpatient and outpatient costs,^25^ combined with updated estimates for salary cost of specific health workers.^26^ The recent updates to the price databases used by WHO-CHOICE have overall higher cost predictions than previous database.^24-26^ All prices are presented here in 2010 International Dollars (2010 was chosen as the baseline year to align with the 2010 Global Burden of Disease study epidemiological data).

Table 2 provides information on assumptions used for target population and mode of delivery. The Supplementary file 2 provides additional detail on cost inputs – including average outpatient visits, health worker time, and health products, per intervention. Costs were estimated for each country using country-specific prices in 2010 I$ and then combined into an aggregate cost for each region, then divided by the total population per sub-region, across 100 years.

###  Comparing Interventions

All interventions and packages were individually compared to the hypothetical “null” scenario in which the effects of all currently implemented interventions are removed.^10^ Health impacts and costs are thus calculated as the difference between the scale-up and null scenarios. All costs and impacts are assessed over a 100-year time frame from 2010-2110, with year-by year results being generated. The average cost-effectiveness ratios (ACERs) were calculated by dividing the total cost for scale-up by the total health gain.

In the main scenario presented here, costs are discounted at 3% per annum, whereas HLYs are not discounted (0% discount rate for impact).^10^ We also analyzed results when costs and HLYs are both discounted at 3% (results in Supplementary file 3). Additional sensitivity analysis was performed through varying the coverage rates and applying one-way deterministic sensitivity analysis of 25% higher or lower costs for medicines and medical supplies.

 Designing a package will require prioritization within a budget constraint. The marginal addition of interventions and packages is explored in order to describe an “expansion path” for an essential benefit package for MNCH impact. The expansion path describes the order in which interventions should be implemented in order to maximize health outcomes for any given budget, assuming that cost-effectiveness is the only criteria considered, and no system constraints. Here, we assess how an expansion path might be constructed in a hypothetical setting in South East Asia. For clarity, we include only interventions at 95% coverage, and apply a maximum budget of 4 million I$. We adjusted impact and costs in cases where previous interventions on the expansion path already captured some of the expected health gains.

## Results

ACERs for 95% coverage are presented in tables 3 and 4. Cost-effectiveness ratios decrease as coverage levels increase from 50% to 80% and 95% (see Supplementary file 3 for results), reflecting economies of scale built into the programme costs.^24^ In general, ACERs are much higher in the South East Asia region than in SSA-E. However, within each region there are interventions which represent very good value for money (Tables 3 and 4).

**Table 3:** Interventions Presented in Bands of Cost-Effectiveness, SSA-E (95% Population Coverage, 3% Discount Rate for Costs, 0% Discount Rate for Health Effects)

**Intervention**	**Short Name**	**ACER**	**Cost Per 1 Million Population (I$)**	**HLY Per 1 Million Population**	**Target Population Group**
<$10/HLY gained					
Skilled assistance for normal delivery + family planning	MNCH_P4. SBA + FP	1.2	6266654	5192430	Pregnant women
Family planning	MNCH_1. FP	2.7	14131612	5256634	Pregnant women
Skilled delivery + management of complications + family planning	MNCH_P6. SBA + comp + FP	0.4	22857472	54115655	Pregnant women
Preventing and managing unplanned pregnancy	MNCH_P1. UPP	0.7	1802557	2523029	Pregnant women
Neonatal resuscitation	MNCH_14. NR	1.0	134391	131675	Newborn
Community-based management of pneumonia	MNCH_34. CCM_P	2.5	154459	61116	Child
Facility-based management of pneumonia	MNCH_36. FCM_P	3.5	210934	61116	Child
Case management of severe neonatal infection (sepsis/pneumonia) with full supportive care	MNCH_31. CMSNI	3.6	149142	41339	Newborn
Vitamin A supplementation (0-4 years)	MNCH_21. VAS	7.1	242300	34309	Child
Facility-based management of neonatal infection (sepsis/pneumonia) with injectable (and oral) antibiotics	MNCH_32. CMNI	8.2	142418	17303	Newborn
Between $10 and <$100/HLY gained					
Measles vaccine	MNCH_28. MCV	10.1	200492	19891	Child
Home visits for clean postnatal practices	MNCH_20. CPNP	11.5	215967	18699	Newborn
Infant and young child feeding	MNCH_P9. IYCF	11.7	629808	53789	Child
Primary level integrated management of the sick child (includes link to the community)	MNCH_P12. IMCI	12.3	1820209	147912	Child
Community-based newborn and child care	MNCH_P8. CBNCC	13.8	2434145	176074	Child
Case management of newborn complications at referral level	MNCH_P7. CMNC	14.4	979674	68096	Newborn
Routine EPI (measles, diphtheria, pertussis, tetanus, and tuberculosis)	MNCH_P10. EPI	14.4	469958	32672	Child
Management of children with severe acute malnutrition	MNCH_37. CMSAM	16.5	234943	14233	Child
H. influenzae b vaccine	MNCH_24. HIB	17.5	650325	37210	Child
Kangaroo mother care	MNCH_29. KMC	20.1	249627	12411	Newborn
Routine EPI + additional vaccines (rotavirus, pneumococcal, HepB – if we use the pentavalent )	MNCH_P11. EPI + ROTA + PCV	20.1	1023615	51010	Child
Pentavalent vaccine (DPT + Hep B + Hib)	MNCH_27. PENTA (DPT + HEPB + HIB)	20.1	296640	14791	Child
Management of diarrhea through oral rehydration solution and zinc	MNCH_33. ORSzinc	22.3	1818802	81557	Child
Tetanus toxoid vaccination	MNCH_8. TT	22.6	227810	10073	Pregnant women
Clean cord care (clean birth practices)	MNCH_15. CCC	23.8	137059	5759	Newborn
Syphilis detection and treatment in pregnancy	MNCH_10.SYP	24.8	233088	9417	Pregnant women
Comprehensive antenatal care	MNCH_P2. ANC	26.8	1019342	37988	Pregnant women
Balanced energy-protein supplementation to pregnant women with insecure food availability	MNCH_7. BEPS	27.9	427704	15336	Pregnant women
Promotion of breastfeeding	MNCH_19. BF	29.0	331717	11449	Newborn
Skilled assistance for normal delivery	MNCH_P3. SBA	29.6	4558206	153977	Pregnant women
Rotavirus vaccine	MNCH_26. ROTA	30.1	386284	12840	Child
Pneumococcal vaccine	MNCH_25. PCV	34.9	750344	21498	Child
Promotion of complementary feeding	MNCH_22. CF	36.7	215932	5882	Child
Intermittent presumptive treatment of malaria	MNCH_9. IPTM	53.7	201762	3755	Pregnant women
Skilled delivery + management of complications	MNCH_P5. SBA + comp	56.9	12423164	218180	Pregnant women
Full supportive care for premature babies	MNCH_30. FSC	62.7	726906	11593	Newborn
Management of pre-eclampsia (mild and severe)	MNCH_12. MPE	85.4	146842	1720	Pregnant women
Management of maternal sepsis	MNCH_18. MMS	93.0	203655	2190	Pregnant women
Hypertensive disease case management in pregnancy	MNCH_11. CMHD	94.9	135050	1424	Pregnant women
Between $100 and < $1000/HLY gained					
Daily iron and folic acid supplementation in pregnant women	MNCH_6. DIFA	111.2	247292	2224	Pregnant women
DPT vaccine	MNCH_23. DPT	111.9	432267	3862	Child
Antibiotics for treatment of dysentery	MNCH_35. DYS	112.7	374913	3325	Child
Safe abortion services	MNCH_3. SA	144.1	151490	1051	Pregnant women
Antibiotics for preterm premature rupture of membranes	MNCH_16. PPROM	184.2	163204	886	Pregnant women
Post abortion case management	MNCH_4. PAC	197.5	155505	787	Pregnant women
Management of eclampsia with magnesium-sulphate	MNCH_17. MEMS	293.9	189484	645	Pregnant women
Folic acid supplementation	MNCH_2. FAS	355.9	191051	537	Pregnant women
Between $1000 and < $10000/HLY gained					
Ectopic pregnancy case management	MNCH_13.ECT	1156.2	160480	139	Pregnant women
Calcium supplementation in pregnant women for the prevention and management of pre-eclampsia/eclampsia	MNCH_5. CS	1310.6	541387	413	Pregnant women

Abbreviations: SSA-E, Eastern sub-Saharan Africa; ACER, average cost-effectiveness ratio; HLYs, healthy life years; EPI, Expanded Programme on Immunization; Hib, Haemophilus influenza type b; DPT, diptheria, tetanus toxoids and pertussis.

**Table 4:** Interventions Presented in Bands of Cost-Effectiveness, SEA (95% Population Coverage, 3% Discount Rate for Costs, 0% Discount Rate for Health Effects)

**Intervention**	**Short Name**	**ACER**	**Cost Per 1 Million Population (I$)**	**HLY Per 1 Million Population**	**Target Population Group**
< $10/HLY gained					
Skilled assistance for normal delivery + family planning	MNCH_P4. SBA + FP	22.1	231276	3256415	Pregnant women
Neonatal resuscitation	MNCH_14. NR	1.7	43712	25726	Newborn
Skilled delivery + management of complications + family planning	MNCH_P6. SBA + comp + FP	35.7	242103	3356485	Pregnant women
Community-based management of pneumonia	MNCH_34. CCM_P	5.0	49649	9890	Child
Case management of severe neonatal infection (sepsis/pneumonia) with full supportive care	MNCH_31. CMSNI	6.6	65864	10035	Newborn
Between $10 and <$100/HLY gained					
Facility-based management of pneumonia	MNCH_36. FCM_P	10.3	101727	9890	Child
Family planning	MNCH_1. FP	11.2	2334143	207711	Pregnant women
Vitamin A supplementation (0-4 years)	MNCH_21. VAS	13.3	97569	7349	Child
Measles vaccine	MNCH_28. MCV	15.6	110365	7085	Child
Home visits for clean postnatal practices	MNCH_20. CPNP	19.0	80671	4241	Newborn
Facility-based management of neonatal infection (sepsis/pneumonia) with injectable (and oral) antibiotics	MNCH_32. CMNI	19.7	51321	2606	Newborn
Preventing and managing unplanned pregnancy	MNCH_P1. UPP	23.0	2394085	104074	Pregnant women
Routine EPI (measles, diphtheria, pertussis, tetanus, and tuberculosis)	MNCH_P10. EPI	30.6	298776	9755	Child
Routine EPI + additional vaccines (rotavirus, pneumococcal, Hep B – if we use the pentavalent)	MNCH_P11. EPI + ROTA + PCV	38.9	491718	12636	Child
Community-based newborn and child care	MNCH_P8. CBNCC	39.8	1026101	25773	Child
Kangaroo mother care	MNCH_29. KMC	44.6	173481	3889	Newborn
Infant and young child feeding	MNCH_P9. IYCF	47.5	498603	10501	Child
Clean cord care (clean birth practices)	MNCH_15. CCC	49.2	43504	885	Newborn
Management of children with severe acute malnutrition	MNCH_37. CMSAM	53.6	107652	2007	Child
Primary level integrated management of the sick child (includes link to the community)	MNCH_P12. IMCI	54.2	905747	16697	Child
Promotion of breastfeeding	MNCH_19. BF	54.6	135622	2482	Newborn
Management of diarrhea through oral rehydration solution and zinc	MNCH_33. ORSzinc	63.3	575694	9102	Child
Case management of newborn complications at referral level	MNCH_P7. CMNC	67.6	847152	12534	Newborn
Pentavalent vaccine (DPT + Hep B + Hib)	MNCH_27. PENTA (DPT + HEPB + HIB)	74.3	167531	2254	Child
Balanced energy-protein supplementation to pregnant women with insecure food availability	MNCH_7. BEPS	87.3	81532	934	Pregnant women
H. influenzae b vaccine	MNCH_24. HIB	90.0	429887	4778	Child
Between $100 and < $1000/HLY gained					
Syphilis detection and treatment in pregnancy	MNCH_10.SYP	102.7	158103	1539	Pregnant women
Promotion of complementary feeding	MNCH_22. CF	109.9	122560	1115	Child
Comprehensive antenatal care	MNCH_P2. ANC	125.9	705147	5600	Pregnant women
Skilled assistance for normal delivery	MNCH_P3. SBA	128.6	3030550	23565	Pregnant women
Tetanus toxoid vaccination	MNCH_8. TT	136.7	155763	1139	Pregnant women
Pneumococcal vaccine	MNCH_25. PCV	138.1	468206	3391	Child
Intermittent presumptive treatment of malaria	MNCH_9. IPTM	142.6	113448	795	Pregnant women
Rotavirus vaccine	MNCH_26. ROTA	149.6	266987	1785	Child
Full supportive care for premature babies	MNCH_30. FSC	154.9	561212	3624	Newborn
Skilled delivery + management of complications	MNCH_P5. SBA + comp	199.7	6869115	34392	Pregnant women
Daily iron and folic acid supplementation in pregnant women	MNCH_6. DIFA	236.1	166450	705	Pregnant women
Folic acid supplementation	MNCH_2. FAS	281.2	104296	371	Pregnant women
Hypertensive disease case management in pregnancy	MNCH_11. CMHD	307.4	42471	138	Pregnant women
Management of pre-eclampsia (mild and severe)	MNCH_12. MPE	342.7	56815	166	Pregnant women
DPT vaccine	MNCH_23.DPT	556.9	349890	628	Child
Antibiotics for treatment of dysentery	MNCH_35. DYS	581.5	230594	397	Child
Management of eclampsia with magnesium-sulphate	MNCH_17. MEMS	733.0	90613	124	Pregnant women
Safe abortion services	MNCH_3. SA	854.5	68083	80	Pregnant women
Post abortion case management	MNCH_4. PAC	875.3	54443	62	Pregnant women
Management of maternal sepsis	MNCH_18. MMS	928.0	99984	108	Pregnant women
Between $1000 and <$10000/HLY gained					
Antibiotics for preterm premature rupture of membranes	MNCH_16. PPROM	1863.3	73422	39	Pregnant women
Calcium supplementation in pregnant women for the prevention and management of pre-eclampsia/eclampsia	MNCH_5. CS	8353.4	364785	44	Pregnant women
Ectopic pregnancy case management	MNCH_13.ECT	9834.5	62928	6	Pregnant women

Abbreviations: SEA, South East Asia; ACER, average cost-effectiveness ratio; HLYs, healthy life years; EPI, Expanded Programme on Immunization; Hib, Haemophilus influenza type b; DPT, diptheria, tetanus toxoids and pertussis.

 In SSA-E, 27 single interventions and all 12 packages have ACERs below I$100, with 8 interventions between $100-$400 and 2 interventions above $1000.

 In SEA, half (26) of the interventions and packages have ACERs below I$100, whereas 21 interventions demonstrate ACERs between I$100-I$1000 and 3 interventions fall above I$1000.

 Generally, the best performing interventions are consistent across the two regions, and include:

- Family planning
- Neonatal resuscitation
- Management of pneumonia
- Vitamin A supplementation
- Management of neonatal infection (sepsis/pneumonia)
- Measles vaccine

Across both regions, ACERs below I$100 can be found across all delivery platforms, from community to hospital level. It should be noted that all interventions classified here as “community” have ACERs below I$100.

A comparison across countries and programme areas reveals that, out of the interventions analysed, child health and immunization produce the most favourable ACERs. Across the life course, interventions targeting the newborn have the lowest ACERs, closely followed by interventions targeting under-fives (Table 5). In terms of single interventions across the life course, the 9 newborn health interventions are among the most cost-effective, with ACERs ranging from 1.0 to 154.9 across the 2 regions (median = 14.4). Next, child interventions ACERs are estimated to range between 2.5 and 581.5 (median = 15.4). Finally, interventions delivered during pregnancy and child birth have ACERs which range from 0.3 to 9834.5 (median = 27.4). Two interventions come out as the least cost-effective across the two regions: calcium supplementation in pregnant women, and ectopic pregnancy case management.

**Table 5:** Summary Results by Programme and Life Course Approach (Interventions and Packages Evaluated at 95% Coverage): Comparison of ACERs Across All Countries Included in Study

	**No. of Interventions**	**Average ACER**	**Lowest ACER**	**Highest ACER**
SSA-E				
Maternal and newborn health (programme)	26	100.5	0.3	1156.2
Child health (programme)	6	27.9	2.5	112.7
Immunization (programme)	8	32.4	10.1	111.9
Nutrition (programme)	9	211.8	7.1	1310.6
Pregnant women/women of reproductive age (life course)	22	194.1	0.3	1310.6
Newborn (life course)	9	19.4	1.0	62.7
Children aged 1-59 months (life course)	18	27.7	2.5	112.7
SEA				
Maternal and newborn health (programme)	26	655.0	1.7	9834.5
Child health (programme)	6	125.7	5.0	581.5
Immunization (programme)	8	136.8	15.6	556.9
Nutrition (programme)	9	1026.3	13.3	8353.4
Pregnant women/women of reproductive age (life course)	22	1164.8	11.2	9834.5
Newborn (life course)	9	46.4	1.7	154.9
Children aged 1-59 months (life course)	18	115.1	5.0	581.5

Abbreviations: ACER, average cost-effectiveness ratio; SEA, South East Asia; SSA-E, Eastern sub-Saharan Africa.

 Overall, the combination of interventions into packages produces favorable ACERs. An example is antenatal care (P2) where the package fares better than individual components such as hypertensive disease case management in pregnancy. The reason for this is the modelled economies of scale introduced in combining facility visits and programme costs.

**Figure 1:**
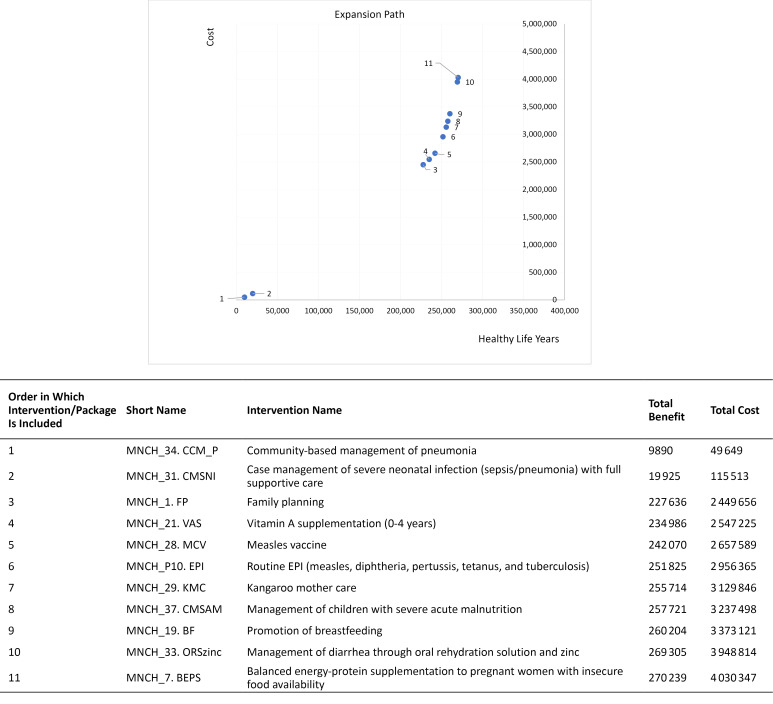
The Design of an Expansion Path for SEA: An Illustrative Example (Considering Interventions at 95% Coverage). Abbreviations: SEA, South East Asia; EPI, Expanded Programme on Immunization.

The design of an expansion path for SEA is illustrated in Figure 1. The first intervention is community based management of pneumonia, with an ACER of 5.0; at a cost of 49649 and 9890 HLYs gained. The second intervention included is Case management of severe neonatal infection (sepsis/pneumonia) with full supportive care. Adding subsequent interventions pushes costs upwards until the budget constraint of $4 million is reached. Under these constraints, a total of 11 interventions and packages would be included, if cost-effectiveness was the main criteria. Most interventions included target newborn and child health outcomes.

When a 3% discount rate was applied to benefits, ACERs were significantly higher, indicating that each HLY now came at a higher cost (Supplementary file 3). Interventions and packages that include family planning were pushed a few steps down the ranks, since the effects appear further down the time horizon Still, they remain important interventions, but now somewhat less dominant in the rank order. Aside from this effect, the rank ordering of interventions did not change. Similarly, when costs for commodities and supplies were reduced or increased by 25%, the rank order did not change – indicating that drug and supply inputs are not cost drivers.

**Figure 2:**
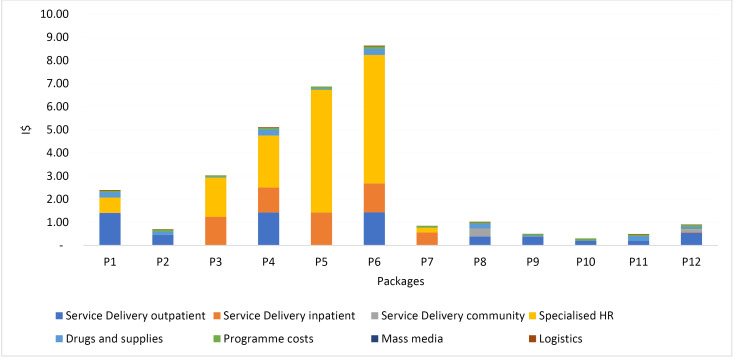
Annual Cost Per Capita, 12 Packages, by Cost Component (at 95% Coverage, South East Asia region).

A breakdown of costs can be useful to examine cost drivers. Figure 2 provides estimates of the annual economic cost of providing the 12 packages, per capita, in I$, in the region of SEA. Specialized health work force is an important contributor to cost for packages P3-P6; less so for the other packages.

## Discussion

 We have presented updated WHO-CHOICE results for interventions targeting MNCH outcomes in two geographic regions, as part of a broader update of WHO-CHOICE cost-effectiveness estimates. Examining interventions at 95% coverage, results for SSA-E indicate that more than 39 intervention/package options are available which cost less than $100 per healthy life year gained, with an additional 10 options under $1000 per HLY gained (3% discount rate for costs; 0% discount rate for outcomes). In SEA, overall costs are higher and thus ACERs are in general higher than for SSA-E. Still, 26 options cost less than $100 per healthy life year and an additional 21 are available for ACERs less than $1000.

 Cost-effective interventions for MNCH can be found in all dimensions of a health system. First, we note that the I$ 0-10 category includes interventions delivered at all platforms, from community level up to primary level and up to hospital level. It is therefore not a given that lower level service delivery platforms should be prioritized on the basis of cost-effectiveness, although other reasons may point in that direction, such as health workforce constraints. Second, cost-effective interventions exist across the life course and cover both prevention and curative interventions. Access to contraceptives through family planning stands out as an investment with high value for money. The counterfactual for the family planning intervention is a context where no-one has access to contraceptives, not even through purchase in pharmacies, which is why the model produces highly cost-effective results. Third, we note the high cost-effectiveness of integrated packages across programmatic areas, including nutrition, immunization, and management of risks, such as within comprehensive antenatal care. Package options are more cost-effective than single procedures around birth (eg, management of eclampsia with magnesium-sulphate), and packages of care are also more feasible in terms of programme implementation.

 Similarities in rank order across the two regions are driven by the fact that both regions have high maternal and infant mortality, and that many interventions bring consistent value for money across settings – such as management of pneumonia and routine immunization. Indeed, we would not expect otherwise. However, there are important differences across regions. An example is management of maternal sepsis which is given a higher ranking in SSA than in SEA, due to the underlying burden. Across settings, there will be differences in epidemiological structure, related social and economic determinants, commodity prices, costs of health workforce and other inputs, that warrant the need for a context-specific analysis. For this analysis we have compared target coverages against a null scenario. At country level, it would be useful to also compare target coverage against current coverage, in order to assess how far off current investments are from the idealized expansion path.

Our findings are consistent with the published literature, which has previously demonstrated high cost-effectiveness of many interventions targeting MNCH outcomes.^27^ However, most existing publications are restricted to individual interventions, and do not compare across interventions and packages. Moreover, there is considerable variation across studies in terms of the settings/context (related to country epidemiology and delivery mechanisms), and the analytical methods used (such as time frame and discount rates). For example, many analyses do not report shared health systems costs.^27^ Efforts made by initiatives such as DCP3 (Disease Control Priorities, third edition) to consolidate cost-effectiveness evidence are important to the extent that they provide a landscape of the published literature, however they suffer from limitations since they compare studies that use different methods and assumptions.^27^ To our knowledge, the WHO-CHOICE approach is unique in generating new estimates for interventions across a range of health programmes through the use of a standardized methodological framework, which explicitly identifies and estimates shared health system costs at and above facility level.

Here we present normative estimates for specific geographic regions (“normative” referring to estimates generated for a setting with well-functioning health systems, and where best practice is followed). While there is considerable uncertainty with respect to estimates for the cost per HLY gained, the overall findings are consistent with previous analysis^13,14^ as we continue to find that community and facility-based newborn care, vitamin A supplementation and measles vaccine rank among the most cost-effective interventions. The most striking difference from our updated analysis is the demonstrated high value of family planning. Family planning may be regarded as a distal intervention for reducing maternal mortality as compared to clinical care during pregnancy and childbirth, however our analysis demonstrates that at population level, contraception can play an important role for mortality reduction.

Differences in intervention-specific cost-effectiveness estimates compared to the prior analysis are driven by changes in the underlying model (LiST compared to prior Excel based model) and methods (new WHO-CHOICE analysis has adopted approaches where the main scenario presented does not discount health benefits, and also lengthened the implementation period over which health benefits are modelled). While efficacy estimates have not drastically changed, the price databases used by WHO-CHOICE have been updated and costs are now estimated to be higher than in previous studies. In particular, within this analysis we have sought to specifically account for costs related to specialized health workforce, using country-specific salary estimates.^26^Figure 2 demonstrated that packages that entail specific health workforce have higher costs than packages which do not require such resource. On the other hand, commodity costs are modest in comparison. This is also due to falling vaccine prices in recent years. These results underline the need to consider affordability and system constraints when prioritizing interventions for benefit packages.

The interventions analyzed conform to WHO guidelines. Our analysis shows that many interventions recommended by WHO are highly cost-effective, but some interventions less so. This underpins the need to consider economic analysis and resource implications within the guideline development process. An example is the WHO 2011 Calcium supplementation guideline which was revalidated in 2018, also in the context of the antenatal care. At the time, resources required for implementation were judged as high compared with other supplements such as iron and folate, and the cost-effectiveness was described as “unknown.”^28^ Here we present results that confirm the relatively high cost for implementing calcium supplementation alone, as it ranks last in both regions, though the ANC package (which included calcium supplementation) produced favorable ACERs.

###  Limitations

The most concerning limitation in our model is the focus on mortality outcomes, with less consideration of morbidity and overall well-being. Most interventions act on risks associated with acute events and with high mortality risks. This focus is driven by current evidence. Our analysis draws on the existing tool set for impact modelling within the Spectrum platform, which would benefit from further expansion. The LiST tool does not fully incorporate all WHO guidelines and not all relevant interventions. There are however current efforts ongoing to address these issues and expand the Spectrum platform to enable modelling a broader set of actions and outcomes, including an expanded set of essential nutrition interventions.^29^

 Furthermore, we undertook limited uncertainty analysis. Many interventions have similar ACERs, and adjusting one or more variables could change the relative order of ranking. The expansion path presented here should therefore not be interpreted as absolute, but as an indicative example of how a country could examine the order in which to expand the coverage for different interventions.

## Conclusion

 Most interventions in our analysis are already being delivered in LMICs, and there is currently considerable variation in service uptake across interventions; while immunization rates are generally high, reported coverage of pneumonia treatment lags behind.

We argue that, in a context of decreasing development assistance for health, the MNCH agenda is still vulnerable.^30^ Evidence on the cost-effectiveness of interventions to improve MNCH outcomes must continuously be emphasized to ensure that resources are allocated to support their implementation. Beyond cost-effectiveness, criteria to consider include targeting the vulnerable, but also overall system capacity to expand coverage, and the absolute levels of investment (financing) needed for expanding service coverage. In order to enable and encourage country-level analysis that uses local data, WHO has shifted its tool set to the Spectrum-based platform which allows for such considerations. Here countries can conduct cost-effectiveness analysis using the Spectrum cost-effectiveness tool and then assess health system implications and financial costs, using the OneHealth Tool, in both cases using the same set of impact models and applying local data and assumptions (see https://www.who.int/teams/health-systems-governance-and-financing/economic-analysis).

It should be emphasized that, while cost-effectiveness can help identify value for money, the achievement of the SDG mortality targets requires investing in packages beyond the most easily implemented “best buys.” Previous research has underlined that most MNCH-related deaths will be prevented by quality care provided at facility level.^31^ Reducing maternal and newborn mortality to achieve the 2030 targets will require accessible and good quality clinical services. Moreover, investments in other sectors – such as housing, agriculture, energy and education—is critical.^32^

 With maternal and child mortality still looming high in many countries, there are opportunities to gear investments towards high-impact interventions. Evidence on cost-effectiveness can inform national processes on what to include in the benefit package from a universal health coverage perspective. These tools can be used at national level to inform the design of benefit packages, GFF investment cases and overall priority setting processes.

## Ethical issues

 No ethical approval was sought as this is a secondary data analysis.

## Competing interests

 Authors declare that they have no competing interests.

## Authors’ contributions

 KS conceptualized the paper together with RW and MYB. KS and RW set up the models, conducted the analysis, and validated the results. All authors analyzed and interpreted the results. KS drafted the first version of the manuscript. All authors critically reviewed and edited the manuscript.

## Authors’ affiliations

^1^Department of Health Systems Governance and Financing, World Health Organization (WHO), Geneva, Switzerland. ^2^School of Population and Global Health, The University of Western Australia, Crawley, WA, Australia. ^3^Department of Nutrition for Health and Development, World Health Organization (WHO), Geneva, Switzerland. ^4^Department of Maternal, Newborn, Child and Adolescent Health and Ageing, World Health Organization (WHO), Geneva, Switzerland. ^5^Department of Sexual and Reproductive Health and Research, World Health Organization (WHO), Geneva, Switzerland. ^6^Department of Immunization, Vaccines and Biologicals (IVB), World Health Organization (WHO), Geneva, Switzerland.

## Supplementary files


Supplementary file 1. Effect Sizes Used Within Analysis.

Supplementary file 2. Details on Cost Inputs and Prices Used in Analysis.

Supplementary file 3. Cost-Effectiveness Results by Level of Coverage, and With Varied Discount Rates.
