# Supplementary material for: Cost-Effectiveness of Interventions to Improve Maternal, Newborn and Child Health Outcomes: A WHO-CHOICE Analysis for Eastern Sub-Saharan Africa and South-East Asia
Source: Int J Health Policy Manag. 2021 Mar 17;10(11):706–23. doi: 10.34172/ijhpm.2021.07 (PMC9278381; doi:10.34172/ijhpm.2021.07)
Supplement: Supplementary file 1 — Effect Sizes Used Within Analysis. [file ijhpm-10-706-s001.pdf]

Cost-Effectiveness of Interventions to Improve Maternal, Newborn and Child Health Outcomes: A WHO-CHOICE Analysis for Eastern Sub-Saharan Africa and South-East Asia
International Journal of Health Policy and Management (IJHPM)
Authors: Karin Stenberg*, Rory Watts, Melanie Bertram, Kaia Engesveen, Blerta Maliqi, Lale Say, Raymond Hutubessy
*Corresponding author: stenbergk@who.int

**Supplementary file 1.** Effect Sizes Used Within Analysis

| No | Single Interventions | Target For Impact | Impact Size | Impact Reference |
|---|---|---|---|---|
| 1 | Family planning | Maternal mortality | Family planning increases the age at first birth, and subsequent birth intervals. Effectiveness is measured as the proportion of users who do not become pregnant during a year of method use. Within the tool they range from 81-100%. Impact is calculated as the difference in all cause maternal deaths between the scale-up scenario and the Null scenario (both scenarios with the same baseline MMR ). | Stover, John, Jane T. Bertrand, Susan Smith, Naomi Rutenberg and Kimberly Meyer-Ramirez. 1997 Empirically Based Conversion Factors for Calculating Couple-Years of Protection . ChapelHill,NC: The EVALUATION Project. |
| 2 | Folic acid supplementation | Newborn (0-1 month) | Effect size 0.62. Affected Fraction 0.29. | Blencowe H, Cousens S, Modell B, Lawn J. Folic acid to reduce neonatal mortality from neural tube disorders. International Journal of Epidemiology. 2010;39(Suppl 1):i110-i121. doi:10.1093/ije/dyq028. |
| | | Stillbirths | Effect size 0.41. Affected Fraction 0.02. | Imdad A, Yakoob MY, Bhutta ZA. The effect of folic acid, protein energy and multiple micronutrient supplements in pregnancy on stillbirths. BMC Public Health 2011; 11(Suppl 3):S4. |
| 3 | Safe abortion services | Maternal mortality | Effect on maternal mortality due to abortion. Effectiveness: 0.95 Affected Fraction: 0.905 | Pollard SL, Mathai M, Walker N. Estimating the impact of interventions on cause-specific maternal mortality: a Delphi approach. BMC Public Health. 2013;13(Suppl 3):S12. doi:10.1186/1471-2458-13-S3-S12. |
| 4 | Post abortion case management | Maternal mortality | Effect on maternal mortality due to abortion. Effectiveness: 0.8 Affected Fraction: 0.905 | Pollard SL, et al (as above). |

| | | | | |
|---|---|---|---|---|
| 5 | Calcium supplementation in pregnant women for the prevention and management of pre-eclampsia/eclampsia | Maternal mortality | Effect on maternal mortality due to hypertensive disorders. Effectiveness: 0.2 Affected Fraction: 1.0 | Jabeen M, Yakoob MY, Imdad A, et al. Impact of interventions to prevent and manage preeclampsia and eclampsia on stillbirths. BMC Public Health 2011; 11(Suppl 3): S6. http://www.ncbi.nlm.nih.gov/pmc/articles/PMC3231912/.<br><br>Ronsmans C, Campbell O. Quantifying the fall in mortality associated with interventions related to hypertensive diseases of pregnancy. BMC Public Health 2011; 11(Suppl 3): S8. https://www.ncbi.nlm.nih.gov/pmc/articles/PMC3231914/.<br><br>Imdad A, Jabeen A, Bhutta, ZA. Role of calcium supplementation during pregnancy in reducing risk of developing gestational hypertensive disorders: a meta-analysis of studies from developing countries. BMC Public Health 2011; 11(Suppl 3): S18. https://www.ncbi.nlm.nih.gov/pmc/articles/PMC3231891/. |
| 6 | Daily iron and folic acid supplementation in pregnant women | Newborn (0-1 month) | Effect on newborn mortality due to congenital anomalies. Effectiveness 0.46. Affected Fraction 0.20 | Haider BA, Yakoob MY, Bhutta ZA. Effect of multiple micronutrient supplementation during pregnancy on maternal and birth outcomes. BMC Public Health 2011; 11(Suppl 3): S19. http://www.ncbi.nlm.nih.gov/pmc/articles/PMC3231892/. |
| 7 | Balanced energy-protein supplementation to pregnant women with insecure food availability | Newborn (0-1 month) | Effect on antepartum and intrapartum stillbirths: Effectiveness: 0.4. Affected Fraction: country-specific | Imdad A, Bhutta ZA. Effect of balanced protein energy supplementation during pregnancy on birth outcomes. BMC Public Health 2011; 11(Suppl 3): S17. http://www.ncbi.nlm.nih.gov/pubmedhealth/PMH0031506<br><br>Ota E, Hori H, Mori R, et al. Antenatal dietary education and supplementation to increase energy and protein intake. Cochrane Database Syst Rev 2015. http://www.ncbi.nlm.nih.gov/pubmed/26031211.<br><br>Jackson BD, Walker N, Heidkamp R. Metrics for Identifying Food Security Status and the Population with Potential to Benefit from Nutrition Interventions in the Lives |

| | | | | |
|---|---|---|---|---|
| | | | | Saved Tool (LiST). J Nutrition 2017, 147(11S): 2147S-2155S. https://doi.org/10.3945/jn.116.243808 |
| 8 | Tetanus toxoid vaccination | Maternal mortality | Effect on indirect causes: Effectiveness 0.98. Affected fraction 0.005 | Blencowe H, Lawn J, Vandelaer J, et al. Tetanus toxoid immunization to reduce mortality from neonatal tetanus. International Journal of Epidemiology 2010; 39(Suppl 1): i102-i109. http://www.ncbi.nlm.nih.gov/pmc/articles/PMC2845866/. |
| | | Newborn (0-1 month) | Effect on indirect causes: Effectiveness 0.98. Affected fraction 1.0 | |
| 9 | Intermittent presumptive treatment of malaria in pregnancy | Maternal mortality | Effect on indirect causes: Effectiveness 0.725. Affected fraction 0.045 | Eisele TP, Larsen D, Steketee RW. Protective efficacy of interventions for preventing malaria mortality in children in Plasmodium falciparum endemic areaIsn.ternational Journal of Epidemiology 2010; 39(Suppl 1): i88-i10. http://www.ncbi.nlm.nih.gov/pmc/articles/PMC2845865/. (The effect size for ITN/IRS is used as a proxy for IPTp.)<br><br>Pollard SL, Mathai M, Walker N. Estimating the impact of interventions on cause-specific maternal mortality: A Delphi approacBhM. C Public Health 2013; 13(Suppl 3): S12. http://www.ncbi.nlm.nih.gov/pmc/articles/PMC3847442/.<br><br>Ishaque S, Yakoob MY, Imdad A, et al. Effectiveness of interventions to screen and manage infections during pregnancy on reducing stillbirths: A reviewBM. C Public Health 2011; 11(Suppl 3): S3. http://www.ncbi.nlm.nih.gov/pmc/articles/PMC3231903/. (The effect size for ITN usage is used as a proxy for IPTp.)<br><br>Radeva-Petrova D, Kayentao K, Ter Kuile FO, et al. Drugs for preventing malaria in pregnant women in endemic areas: Any drug regimen versus placebo or no treatmenCt.ochrane Database Syst Rev 2014. http://www.ncbi.nlm.nih.gov/pmc/articles/PMC4498495/. |
| | | Stillbirths | Effectiveness 0.33. Affected fraction: country specific | |

| | | | | |
|---|---|---|---|---|
| 10 | Syphilis detection and treatment in pregnancy | Newborn (0-1 month) | Effect on newborn mortality due to sepsis: Effectiveness: 0.97. Affected Fraction: 0.006 | Blencowe H, Cousens S, Kamb M, et al. Lives Saved Tool supplement detection and treatment of syphilis in pregnancy to reduce syphilis related stillbirths and neonatal mortality. BMC Public Health 2011; 11(Suppl 3): S9. http://www.ncbi.nlm.nih.gov/pubmed/21501460. |
| | | Stillbirths | Effect on antepartum stillbirths: Effectiveness: 0.82. Affected Fraction: 0.002 | Blencowe H, Cousens S, Kamb M, et al. Lives Saved Tool supplement detection and treatment of syphilis in pregnancy to reduce syphilis related stillbirths and neonatal mortality. BMC Public Health 2011; 11(Suppl 3): S9. http://www.ncbi.nlm.nih.gov/pubmed/21501460. |
| 11 | Hypertensive disease case management in pregnancy | Maternal mortality | Effect on maternal mortality due to hypertensive disorders. Effectiveness: 0.5 Affected Fraction: 1.0 | Pollard SL, Mathai M, Walker N. Estimating the impact of interventions on cause-specific maternal mortality: A Delphi approach. BMC Public Health 2013; 13(Suppl 3): S12. http://www.ncbi.nlm.nih.gov/pmc/articles/PMC3847442/. |
| 12 | Management of pre-eclampsia (mild and severe) | Maternal mortality | Effect on maternal mortality due to hypertensive disorders. Effectiveness: 0.59 Affected Fraction: 1.0 | Ronsmans C, Campbell O. Quantifying the fall in mortality associated with interventions related to hypertensive diseases of pregnancy. BMC Public Health 2011; 11(Suppl 3): S8. http://www.ncbi.nlm.nih.gov/pubmed/21501459.<br><br>Jabeen M, Yakoob MY, Imdad A, et al. Impact of interventions to prevent and manage preeclampsia and eclampsia on stillbirths. BMC Public Health 2011; 11(Suppl 3): S6. http://www.ncbi.nlm.nih.gov/pmc/articles/PMC3231912/. |
| 13 | Ectopic pregnancy case management | Maternal mortality | Effect on maternal mortality due to abortion. Effectiveness: 0.900 Affected Fraction: 0.184 | Pollard SL, Mathai M, Walker N. Estimating the impact of interventions on cause-specific maternal mortality: A Delphi approach. BMC Public Health 2013; 13(Suppl 3): S12. http://www.ncbi.nlm.nih.gov/pmc/articles/PMC3847442/. |
| 14 | Neonatal resuscitation | Newborn (0-1 month) | Effect on newborn mortality due to asphyxia: Effectiveness: 0.3 at CEmOC*. Affected Fraction: 1.0.<br>Effect on newborn mortality due to prematurity: Effectiveness: 0.1 at CEmOC*. Affected Fraction: 1.0 | Lee AC, Cousens S, Mullany LC, et al. Neonatal resuscitation and immediate newborn assessment and stimulation for the prevention of neonatal deaths: A systematic review, meta-analysis and Delphi estimation of mortality effect. BMC Public Health 2011; 11(Suppl 3): S12. http://www.ncbi.nlm.nih.gov/pubmed/21501429 |

| | | | | |
|---|---|---|---|---|
| 15 | Clean cord care (clean birth practices) | Newborn (0-1 month) | Effect on newborn mortality due to sepsis: Effectiveness: 0.23. Affected Fraction: 1.0. | Blencowe H, Cousens S, Mullany LC, et al. Clean birth and postnatal care practices to reduce neonatal deaths from sepsis and tetanus: A systematic review and Delphi estimation of mortality effect. BMC Public Health 2011; 11(Suppl 3): S11. http://www.ncbi.nlm.nih.gov/pmc/articles/PMC3231884/.<br><br>Pollard SL, Mathai M, Walker N. Estimating the impact of interventions on cause-specific maternal mortality: A Delphi approach. BMC Public Health 2013; 13(Suppl 3): S12. http://www.ncbi.nlm.nih.gov/pmc/articles/PMC3847442/. Top |
| 16 | Antibiotics for preterm premature rupture of membranes. | Maternal mortality | Effect on maternal mortality due to Sepsis. Effectiveness: 0.80 at CEmOC*. Affected Fraction: 0.33 | Cousens S, Blencowe H, Gravett M, et al. Antibiotics for pre-term pre-labour rupture of the membranes: Prevention of neonatal deaths due to complications of preterm birth and infection. International Journal of Epidemiology 2010; 39(Suppl 1): i34-i43. http://www.ncbi.nlm.nih.gov/pubmed/20348116. |
| 17 | Management of eclampsia with Magnesium-Sulphate | Maternal mortality | Effect on maternal mortality due to hypertensive disorders. Effectiveness: 0.60 at CEmOC*. Affected Fraction: 1.0. | Ronsmans C, Campbell O. Quantifying the fall in mortality associated with interventions related to hypertensive diseases of pregnancy. BMC Public Health 2011; 11(Suppl 3): S8. http://www.ncbi.nlm.nih.gov/pubmed/21501459.<br><br>Jabeen M, Yakoob MY, Imdad A, et al. Impact of interventions to prevent and manage preeclampsia and eclampsia on stillbirths. BMC Public Health 2011; 11(Suppl 3): S6. http://www.ncbi.nlm.nih.gov/pubmed/21501457. |
| 18 | Management of maternal sepsis | Maternal mortality | Effect on maternal mortality due to Sepsis. Effectiveness: 0.80. Affected Fraction: 1.0. | Pollard SL, Mathai M, Walker N. Estimating the impact of interventions on cause-specific maternal mortality: A Delphi approach. BMC Public Health 2013; 13(Suppl 3): S12. http://www.ncbi.nlm.nih.gov/pmc/articles/PMC3847442/. |
| 19 | Promotion of breastfeeding | Newborn (0-1 month) and | Effect size reference: Black RE, Victora CG, Walker SP, et al. Maternal and child undernutrition and overweight in low-income and middle-income countries. Lancet 2013; 382(9890): | Relative Risk varies by mode of breastfeeding, for different causes of post-neonatal death (diarrhea, pneumonia, meningitis, measles, pertussis), and by age group 1-5 months, 6-11 months, 12-23 months, and 24-59 months. |

| | | | | |
|---|---|---|---|---|
| | | child (1-59 months) | 427-51. http://www.ncbi.nlm.nih.gov/pubmed/23746772. (Supplementary material - Web Table 17.)<br><br>NEOVITA Study Group. Timing of initiation, patterns of breastfeeding, and infant survival: prospective analysis of pooled data from three randomised trials. Lancet Global Health 2016; 4(4): e266-75. https://www.ncbi.nlm.nih.gov/pubmed/27013313. | |
| 20 | Home visits for clean postnatal practices | Newborn (0-1 month) | Effect on newborn mortality due to tetanus. Effectiveness: 0.4. Affected Fraction: 1.0. | Blencowe H, Cousens S, Mullany LC, et al. Clean birth and postnatal care practices to reduce neonatal deaths from sepsis and tetanus: A systematic review and Delphi estimation of mortality effect. BMC Public Health 2011; 11(Suppl 3): S11. http://www.ncbi.nlm.nih.gov/pmc/articles/PMC3231884/. |
| 21 | Vitamin A supplementation (0-4 years) | Child (1-59 months) | Effect on child mortality >6 months due to Diarrhea. Effectiveness: 0.53. Affected Fraction: 0.335. | Imdad A, Yakoob MY, Sudfeld CR, et al. Impact of vitamin A supplementation on infant and childhood mortality. BMC Public Health 2011; 11(Suppl 3): S20. http://www.ncbi.nlm.nih.gov/pubmed/21501438. |
| 22 | Promotion of complementary feeding | Child (1-59 months) | Odds ratio for stunting: food secure with promotion 1.00 whereas food secure without promotion 1.30. The stunting prevalence further determines relative risk for mortality due to diarrhea, pneumonia, meningitis, measles, and deaths due to Other causes in the LiST model. | Imdad A, Yakoob MY, Bhutta ZA. Impact of maternal education about complementary feeding and provision of complementary foods on child growth in developing countries. BMC Public Health 2011; 11(Suppl 3): S25. http://www.ncbi.nlm.nih.gov/pubmed/21501443.<br><br>Bhutta ZA, Ahmed T, Black RE, et al. What works? Interventions for maternal and child undernutrition and survival. Lancet 2008; 371: 417-40. http://www.ncbi.nlm.nih.gov/pubmed/18206226.<br><br>Olofin I, McDonald CM, Ezzati M, et al. Associations of Suboptimal Growth with All-Cause and Cause-Specific Mortality in Children under Five Years: A Pooled Analysis of Ten Prospective Studies. PLOS One 2013; 8(5): e64636. http://www.ncbi.nlm.nih.gov/pmc/articles/PMC3667136/. |

| | | | | |
|---|---|---|---|---|
| 23 | DPT vaccine | Child (1-59 months) | Effect on newborn mortality due to pertussis: effectiveness 0.84, affected fraction: 1.0 | Fulton TR, Phadke VK, Orenstein WA, et al. Protective Effect of Contemporary Pertussis Vaccines: A Systematic Review and Meta-analysis. Clinical Infectious Diseases 2016; 62(9): 1100-1100. http://www.ncbi.nlm.nih.gov/pubmed/26908803 |
| 24 | H. influenzae b vaccine | Child (1-59 months) | Effect on newborn mortality due to pneumonia Effectiveness: 0.93 Affected Fraction: 0.213 Effect on newborn mortality due to meningitis: Effectiveness: 0.93 Affected Fraction: 0.46 | Fisher Walker CL, Black RE. Rotavirus vaccine and diarrhea mortality: Quantifying regional variation in effect size. BMC Public Health 2011; 11(Suppl 3): S16. http://www.ncbi.nlm.nih.gov/pmc/articles/PMC3231889/ |
| 25 | Pneumococcal vaccine | Child (1-59 months) | Effect on newborn mortality due to pneumonia. Effectiveness: 0.58. Affected Fraction: 0.328. Effect on newborn mortality due to meningitis: Effectiveness: 0.58. Affected Fraction: 0.52. Effect on newborn mortality due to other causes: Effectiveness: 0.58. Affected Fraction: 0.024. | Fisher Walker CL, Black RE. Rotavirus vaccine and diarrhea mortality: Quantifying regional variation in effect size. BMC Public Health 2011; 11(Suppl 3): S16. http://www.ncbi.nlm.nih.gov/pmc/articles/PMC3231889/ |
| 26 | Rotavirus vaccine | Child (1-59 months) | Effect on newborn mortality due to diarrhea. Effectiveness varies by region: (East Asia 0.427), affected fraction: 0.255 | Fisher Walker CL, Black RE. Rotavirus vaccine and diarrhea mortality: Quantifying regional variation in effect size. BMC Public Health 2011; 11(Suppl 3): S16. http://www.ncbi.nlm.nih.gov/pmc/articles/PMC3231889/ |
| 27 | Pentavalent vaccine (DPT + Hep B + Hib) | Child (1-59 months) | See the individual vaccines DPT, Hib, and HepB for effect information | |
| 28 | Measles vaccine | Child (1-59 months) | Effect on newborn mortality due to measles: effectiveness 0.85, affected fraction: 0.255 | Sudfeld CR, Navar AM, Halsey NA. Effectiveness of measles vaccination and vitamin A treatment. International Journal of Epidemiology 2010; 39(Suppl 1): i48-i55. http://www.ncbi.nlm.nih.gov/pubmed/20348126. |
| 29 | Kangaroo mother care (KMC) | Newborn (0-1 month) | Effect on newborn mortality due to prematurity: effectiveness 0.51, affected fraction: 0.58 (the affected fraction is the average percent of premature births that survive to day 3). | Lawn JE, Mwansa-Kambafwile J, Horta BL, et al. 'Kangaroo Mother Care' to prevent deaths due to preterm birth complications. International Journal of Epidemiology 2010; 39(Suppl 1): i44-i54. http://www.ncbi.nlm.nih.gov/pubmed/20348117. Boundy EO, Dastjerdi R, Spiegelman D, et al. Kangaroo mother care and neonatal outcomes: A meta-analysis. Pediatrics 2016; 137(1). http://www.ncbi.nlm.nih.gov/pubmed/26702029. |

| | | | | |
|---|---|---|---|---|
| 30 | Full supportive care for premature babies | Newborn (0-1 month) | Effect on newborn mortality due to prematurity effectiveness 0.80, affected fraction: 1.000 | Bhutta ZA, Das JK, Bahl R, et al. Can available interventions end preventable deaths in mothers, newborn babies, and stillbirths, and at what cosLt?ancet 2014; 384(9940: 347-70. http://www.ncbi.nlm.nih.gov/pubmed/24853604. |
| 31 | Case management of severe neonatal infection (sepsis/pneumonia ) with full supportive care | Newborn (0-1 month) | Effect on newborn mortality due to sepsis: effectiveness 0.80, affected fraction: 1.000; Effect on newborn mortality due to pneumonia effectiveness 0.90, affected fraction: 1.000. | Zaidi AK, Ganatra HA, Syed S, et al. Effect of case management on neonatal mortality due to sepsis and pneumoniBaM. C Public Health 2011; 11(Suppl 3): S13. http://www.ncbi.nlm.nih.gov/pubmed/21501430. |
| 32 | Facility based management of neonatal infection (sepsis/pneumonia ) with injectible (and oral) antibiotics | Newborn (0-1 month) | Effect on newborn mortality due to sepsis: effectiveness 0.65, affected fraction: 1.000; Effect on newborn mortality due to pneumonia effectiveness 0.75, affected fraction: 1.000. | Zaidi AK, Ganatra HA, Syed S, et al. Effect of case management on neonatal mortality due to sepsis and pneumoniBaM. C Public Health 2011; 11(Suppl 3): S13. http://www.ncbi.nlm.nih.gov/pubmed/21501430. |
| 33 | Management of diarrhea through Oral Rehydration Solution and Zinc | Child (1-59 months) | Effect on child mortality due to diarrhea:<br>The effectiveness reported for ORS is 0.93, with affected fraction: 0.88.<br>The effectiveness reported for zinc is 0.23, with affected fraction: 1.00 | Munos M, Fischer Walker CL, Black RE. The effect of oral rehydration solution and recommended home fluids on diarrhea mortalitIyn.ternational Journal of Epidemiology 2010; 39(Suppl 1): i75-i87. http://www.ncbi.nlm.nih.gov/pubmed/20348131.<br><br>Fischer Walker CL, Black RE. Zinc for the treatment of diarrhea: Effect on diarrhea morbidity, mortality and incidence of future episodeIsn.ternational Journal of Epidemiology 2010; 39(Suppl 1): i63-i69. http://www.ncbi.nlm.nih.gov/pubmed/20348128. |
| 34 | Community based management of pneumonia (oral antibiotics) | Child (1-59 months) | Effect on child mortality due to pneumonia: effectiveness 0.7, affected fraction: 1.000. | Theodoratou E, Al-Jilaihawi S, Woodward F, et al. The effect of case management on childhood pneumonia mortality in developing countrieIsn International Journal of Epidemiology 2010; 39(Suppl 1): i155-i171. http://www.ncbi.nlm.nih.gov/pubmed/20348118. |

| | | | | |
|---|---|---|---|---|
| 35 | Antibiotics for treatment of dysentery | Child (1-59 months) | Effect on child mortality due to diarrhea: effectiveness 0.82, affected fraction: 0.12 | Das JK Ali A, Salam RA, et al. Antibiotics for the treatment of Cholera, Shigella and Cryptosporidium in childreBnM. C Public Health 2013; 13(Suppl 3): S10. http://www.ncbi.nlm.nih.gov/pubmed/24564492. |
| 36 | Facility based management of pneumonia (oral antibiotics) | Child (1-59 months) | Effect on child mortality due to pneumonia: effectiveness 0.7, affected fraction: 1.000. | Theodoratou E, Al-Jilaihawi S, Woodward F, et al. The effect of case management on childhood pneumonia mortality in developing countrieIsn International Journal of Epidemiology 2010; 39(Suppl 1): i155-i171. http://www.ncbi.nlm.nih.gov/pubmed/20348118. |
| 37 | Management of children with severe acute malnutrition | Child (1-59 months) | Treatment shifts children from the severely wasted category into the moderately (-3 to -2 Z-score) and mildly (-2 to -1 Zscore) wasted categories. The effectiveness is 0.78. The LiST tool includes a range of fifferent mortality rates for diarrhea, pneumonnia, meningitis, measles, malaria, and other causes, depending on the degree of wasting. Thus, treatment for severe malnutrition shifts children into a category where their risk of mortality from these conditions is lower. | Lenters LM, Wazny K, Webb P, et al. Treatment of severe and moderate acute malnutrition in low- and middle-income settings: A systematic review, meta-analysis and Delphi procesBsM. C Public Health 2013; 13(Suppl 3): S23. http://www.ncbi.nlm.nih.gov/pmc/articles/PMC3847503/.<br><br>Bhutta ZA, Das JK, Rizvi A, et al. Evidence-based interventions for improvement of maternal and child nutrition: what can be done and at what cost? Lancet 2013; 382(9890): 352-77. http://www.ncbi.nlm.nih.gov/pubmed/23746776. (Supplemental material, page 15.) |

Note: All of the impact sizes and Impact references are taken from the OneHealth Tool Manual https://avenirhealth.org/Download/Spectrum/Manuals/OneHealthManualE.pdf (Accessed: 1 March 2020)

Impact size within the LiST tool uses the two concepts of Effectiveness and Affected Fraction. The effectiveness measure refers to the percent of deaths due to a specific cause that are reduced by the intervention. The affected fraction refers to the percent of deaths due to a specific cause which are potentially able to be impacted by a specific intervention.
