# Supplementary material for: Cost-Effectiveness of Interventions to Improve Maternal, Newborn and Child Health Outcomes: A WHO-CHOICE Analysis for Eastern Sub-Saharan Africa and South-East Asia
Source: Int J Health Policy Manag. 2021 Mar 17;10(11):706–23. doi: 10.34172/ijhpm.2021.07 (PMC9278381; doi:10.34172/ijhpm.2021.07)
Supplement: Supplementary file 2 — Details on Cost Inputs and Prices Used in Analysis. [file ijhpm-10-706-s002.pdf]

Cost-Effectiveness of Interventions to Improve Maternal, Newborn and Child Health Outcomes: A WHO-CHOICE Analysis for Eastern Sub-Saharan Africa and South-East Asia
International Journal of Health Policy and Management (IJHPM)
Authors: Karin Stenberg*, Rory Watts, Melanie Bertram, Kaia Engesveen, Blerta Maliqi, Lale Say, Raymond Hutubessy
*corresponding author: stenbergk@who.int

Supplementary file 2. Details on cost inputs and prices used in analysis.

Table S2.1. Cost inputs – including average outpatient visits, health worker time, and health products, per intervention.

Number	Intervention Name	Short name	Intervention Definition	Target population in need of intervention	Health Programme	Service delivery platform	Average number of outpatient visits and inpatient bed days per person (facility level)*	Specialized staff, time * per person receiving the intervention - if applicable	Drugs and supplies (including diagnostics and essential equipment)
	**Single interventions**								
**1**	Family planning	MNCH_1. FP	Women of reproductive age (15-49 years) in union are provided with counseling and information on different methods for contraception. Modern methods include pills and condoms, injectables, IUD, implant, and sterilization, based on country-specific profile of contraceptive methods use.	Women of reproductive age (15-49 years) in union	Maternal and newborn	Primary level care	Varies by country (dependent on method use)	Sterilization methods require on average 69 minutes of time with Other specialist doctor (Ob/Gyn)	Country specific methods mix:<br>Pill - Standard daily regimen<br>Condom - Male<br>Injectable - 3 month (Depo Provera)<br>IUD - Copper-T 380-A IUD (10 years)<br>Implant - Jadelle (5 years)<br>Implant - Sino-Implant (4 years)<br>Female sterilization<br>Male sterilization<br>Vaginal barrier method<br>Vaginal tablets

**2**	Folic acid supplementation	MNCH _2. FAS	All women, from the moment they begin trying to conceive until 12 weeks of gestation, should take a folic acid supplement (400 µg folic acid daily)	Pregnant women	Nutrition	Primary level care, incl. community	2 outpatient visits	-	Ferrous Salt + Folic Acid, tablet, 60 + 0.4 mg
**3**	Safe abortion services	MNCH _3. SA	Safe abortion provided to women seeking to terminate pregnancy. Methods include manual or electric vacuum aspiration and medical abortion (mifepristone followed by a prostaglandin).	Women seeking to terminate pregnancy (incidence of abortion)	Maternal and newborn	Primary level care	1.5 outpatient visits	-	Depending on type of abortion: Lidocaine HCl (in dextrose 7.5%), ampoule 2 ml;<br>Oxytocin, injection, 10 IU in 1 ml ampoule;<br>Syringe, needle + swab;<br>Misoprostol, tablet, 200 mcg;<br>Paracetamol, tablet, 500 mg
**4**	Post abortion case management	MNCH _4. PAC	Treatment of women experiencing complications after undergoing unsafe abortions. Complications include haemorrhage, sepsis, peritonitis, and trauma to the cervix, vagina, uterus, and abdominal organs.	Women seeking to terminate pregnancy (incidence of abortion)	Maternal and newborn	Hospital	2 inpatient bed days	-	Misoprostol, tablet, 200 mcg;<br>Paracetamol, tablet, 500 mg;<br>Ampicillin, powder for injection, 500 mg, vial;<br>Gentamycin, injection, 40 mg/ml in 2 ml vial;<br>Metronidazole, injection, 500 mg in 100 ml vial;<br>Tetracycline, tablet, 500 mg;<br>Foley catheter;<br>Lidocaine HCl (in dextrose 7.5%), ampoule 2 ml;<br>Syringe, needle + swab;<br>Methylergometrine, Injection 0.2 mg/ml, 1 ml amp;<br>Pethidine, 50 mg/ml, 2 ml ampoule;<br>Sodium chloride, injectable solution, 0,9 %, 500 ml
**5**	Calcium supplementation in pregnant women for the prevention and management	MNCH _5. CS	In populations with low dietary calcium intake, daily calcium supplementation (1.5 g–2.0 g oral elemental calcium) is recommended for pregnant women to reduce the risk of pre-eclampsia.	Pregnant women	Nutrition; Maternal and newborn	Primary level care	4 outpatient visits	-	Calcium, tablet, 600 mg

	of pre-eclampsia/eclampsia								
**6**	Daily iron and folic acid supplementation in pregnant women	MNCH _6. DIFA	Daily oral iron and folic acid supplementation with 30 mg to 60 mg of elemental iron and 400 µg (0.4 mg) folic acid is recommended for pregnant women to prevent maternal anaemia, puerperal sepsis, low birth weight, and preterm birth.	Pregnant women	Nutrition; Maternal and newborn	Primary level care, incl. community	4 outpatient visits	-	Ferrous Salt + Folic Acid, tablet, 60 + 0.4 mg
**7**	Balanced energy-protein supplementation to pregnant women living in areas with high food insecurity Balanced	MNCH _7. BEPS	In undernourished populations, balanced energy and protein dietary supplementation is recommended for pregnant women to reduce the risk of stillbirths and small for gestational age neonates.	Pregnant women living in areas with high food insecurity (based on income per capita)	Nutrition; Maternal and newborn	Primary level care, incl. community and outreach	1 outpatient visit	-	Corn Soy Blend for 6 months
**8**	Tetanus toxoid vaccination	MNCH _8. TT	Two injections of tetanus toxoid vaccine.	Pregnant women	Immunization; Maternal and newborn	Primary level care	2 outpatient visits	-	Tetanus toxoid, injection;<br>Syringe, needle + swab
**9**	Intermittent presumptive treatment of malaria in pregnancy	MNCH _9. IPTM	Intermittent presumptive treatment of malaria of all pregnant women living in areas endemic for P. falciparum.	Pregnant women living in areas endemic for P. falciparum.	Malaria; Maternal and newborn	Primary level care	2 outpatient visits	-	Sulfamethoxazole + trimethropin, tablet 400 mg + 80 mg
**10**	Syphilis detection and treatment in pregnancy	MNCH _10. SYP	Screening pregnant women by rapid plasma reagent test and treatment of sero-positive cases with penicillin.	Pregnant women	Maternal and newborn	Primary level care	2 outpatient visits	-	Blood collecting tube, 5 ml;<br>Gloves, exam, latex, disposable, pair;<br>Syringe, needle + swab;<br>Test, Rapid plasma reagin (RPR);

									Benzathine benzylpenicillin, powder for injection, 2.4 million IU;<br>Water for injection, 5 ml ampoule
**11**	Hypertensive disease case management in pregnancy	MNCH _11. CMHD	Management of moderate to severe hypertension without proteinuria	Pregnant women	Maternal and newborn	Primary level care	5 outpatient visits	-	Proteinuria test (dipstick)
**12**	Management of pre-eclampsia (mild and severe)	MNCH _12. MPE	Management of hypertension and mild pre-eclampsia through outpatient care; management of severe pre-eclampsia through with magnesium sulfate through inpatient care	Pregnant women	Maternal and newborn	Primary level care	5 outpatient visits + 0.5 inpatient bed days	-	Bag, urine, collecting, 2000 ml;<br>Foley catheter;<br>Test strips, urine analysis;<br>Hydralazine, powder for injection, 20 mg ampoule;<br>IV giving/infusion set, with needle;<br>Sodium lactate injection (Ringer's), 500 ml, with giving set;<br>Misoprostol, tablet, 200 mcg;<br>Oxytocin, injection, 10 IU in 1 ml ampoule;<br>Sodium chloride, injectable solution, 0,9 %, 500 ml;<br>Magnesium sulfate, injection, 500 mg/ml in 10-ml ampoule;<br>Syringe, needle + swab;<br>Water for injection, 10 ml ampoule;<br>Lidocaine HCl (in dextrose 7.5%), ampoule 2 ml
**13**	Ectopic pregnancy case management	MNCH _13. ECT	Surgical intervention (laparoscopy or laparotomy) to interrupt an ectopic pregnancy.	Pregnant women with ectopic pregnancy	Maternal and newborn	Hospital		240 minutes N/M ; 60 minutes MD; 120 minutes Other specialist doctor	

**14**	Neonatal resuscitation	MNCH _14. NR	Detection of breathing problems and resuscitation of newborn when required, using bag and mask.	Newborn	Maternal and newborn	Primary level care	1 inpatient bed day	20 minutes N/M ; 3 minutes other specialist doctor	Resuscitator,hand-operated.,infant/child,set
**15**	Clean cord care (clean birth practices)	MNCH _15. CCC	Umbilical cord cleansing, with chlorhexidine or other disinfectant.	Newborn	Maternal and newborn	Primary level care	1 outpatient visit	-	Chlorhexidine digluconate (10 ml bottle)
**16**	Antibiotics for preterm premature rupture of membranes.	MNCH _16. PPROM	Hospitalization prior to delivery, with administration of oral antibiotics to women with preterm premature rupture of membranes.	Pregnant women	Maternal and newborn	Hospital	2 inpatient bed days	30 minutes N/M	Amoxicillin, caplet, 250 mg;<br>Erythromycin, tablet, 250 mg
**17**	Management of eclampsia with Magnesium-Sulphate	MNCH _17. MEMS	Management of convulsions associated with eclampsia, occurring ante-, intra- or postpartum.	Pregnant women	Maternal and newborn	Hospital	7 inpatient bed days	1440 minutes N/M ; 60 minutes MD	Bag, urine, collecting, 2000 ml;<br>Foley catheter;<br>Test strips, urine analysis;<br>Hydralazine, powder for injection, 20 mg ampoule;<br>IV giving/infusion set, with needle;<br>Sodium lactate injection (Ringer's), 500 ml, with giving set;<br>Misoprostol, tablet, 200 mcg;<br>Oxytocin, injection, 10 IU in 1 ml ampoule;<br>Sodium chloride, injectable solution, 0,9 %, 500 ml;<br>Magnesium sulfate, injection, 500 mg/ml in 10-ml ampoule;<br>Syringe, needle + swab;<br>Water for injection, 10 ml ampoule;<br>Lidocaine HCl (in dextrose 7.5%), ampoule 2 ml
**18**	Management of maternal sepsis	MNCH _18. MMS	Management of sepsis symptoms within 42 days of delivery.	Pregnant women	Maternal and newborn	Hospital	5 inpatient bed days	60 minutes N/M ; 180	Amoxicillin, caplet, 250 mg;<br>Ampicillin, powder for injection, 500 mg, vial;

								minutes Other staff	Gentamycin, injection, 40 mg/ml in 2 ml vial; Metronidazole, injection, 500 mg in 100 ml vial; Syringe, needle + swab; Water for injection, 5 ml ampoule; Bag, urine, collecting, 2000 ml; Foley catheter; Gloves, surgeon's, latex, disposable, sterile, pair; IV giving/infusion set, with needle; Lancet, blood, disposable; Oxygen, 1000 liters, primarily with oxygen cylinders; Paracetamol, tablet, 500 mg; Sodium chloride, injectable solution, 0,9 %, 500 ml; Complete blood count
**19**	Promotion of breastfeedin g	MNCH _19. BF	Promotion of early and exclusive breastfeeding through skilled care providers and community health workers	Newborn	Nutrition; Maternal and newborn	Primary level care, incl. communi ty	6 outpatient visits	-	
20	Home visits for clean postnatal practices	MNCH _20. CPNP	Home visits within 48 hours of delivery to promote clean practices, specifically that the mother washes her hands frequently, the child lives in a clean environment, and no harmful practices are performed.	Newborn	Maternal and newborn	Commun ity level	1 outpatient visit	-	Clean delivery kit
21	Vitamin A supplementa tion (0-4 years)	MNCH _21. VAS	Vitamin A supplementation for children 6-59 months of age in countries (or sub-national areas in some cases) where vitamin A deficiency is a public health problem	Children 6-59 months	Nutrition; Child	Commun ity level	2 outpatient visits	-	Vitamin A, caplet, 100,000 IU / 200,000 IU

22	Promotion of complementary feeding	MNCH _22. CF	Comprehensive counselling for the caregiver of a child (two full sessions) on the importance of continued breastfeeding after six months of age along with information on appropriate complementary feeding practices, through skilled care providers and community health workers	Children 6-11 months	Nutrition; Child	Primary level care, incl. community	3 outpatient visits	-	
23	DPT vaccine	MNCH _23.DPT	3 doses of DPT vaccine	Newborn	Immunization	Primary level care	3 outpatient visits	-	DTP vaccine;<br>Syringe, auto-disposable, 0.5 ml, with needle;<br>Safety box for used syringes/needles, 5 liter
24	H. influenzae b vaccine	MNCH _24. HIB	3 doses of HiB vaccine	Newborn	Immunization	Primary level care	3 outpatient visits	-	Hib vaccine;<br>Syringe, auto-disposable, 0.5 ml, with needle;<br>Safety box for used syringes/needles, 5 liter
25	Pneumococcal vaccine	MNCH _25. PCV	3 doses of Pneumococcal vaccine	Newborn	Immunization	Primary level care	3 outpatient visits	-	Pneumococcal vaccine;<br>Syringe, auto-disposable, 0.5 ml, with needle;<br>Safety box for used syringes/needles, 5 liter
26	Rotavirus vaccine	MNCH _26. ROTA	3 doses of Rotavirus vaccine	Newborn	Immunization	Primary level care	3 outpatient visits	-	Rotavirus vaccine
27	Pentavalent vaccine (DPT + Hep B + Hib)	MNCH _27. PENTA (DPT + HEPB + HIB)	3 doses of Pentavalent vaccine (a combination of five vaccines-in-one to prevent diphtheria, tetanus, whooping cough, hepatitis b and haemophilus influenza type b).	Newborn	Immunization	Primary level care	3 outpatient visits	-	Pentavalent vaccine (DPT, Hep B, Hib);<br>Syringe, auto-disposable, 0.5 ml, with needle;<br>Safety box for used syringes/needles, 5 liter
28	Measles vaccine	MNCH _28. MCV	2 doses of Measles vaccine	Newborn	Immunization	Primary level care	2 outpatient visits	-	Measles vaccine;<br>Syringe, auto-disposable, 0.5 ml, with needle;<br>Safety box for used syringes/needles, 5 liter

29	Kangaroo mother care (KMC)	MNCH _29. KMC	Inpatient support to Kangaroo Mother Care, defined as continuous skin-to-skin contact between a mother and her newborn as well as frequent and exclusive breastfeeding.	Newborn	Maternal and newborn	Hospital	5 inpatient bed days	30 minutes N/M	
30	Full supportive care for premature babies	MNCH _30. FSC	Prematurely born neonates receive hospital-based full supportive care, including KMC, feeding support/IV fluids, infection prevention/management, oxygen provision, management of neonatal jaundice, nasal CPAP/IPPV (as required), and surfactant for Respiratory Distress Syndrome.	Newborn	Maternal and newborn	Hospital	10 inpatient bed days	1200 minutes N/M ; 600 minutes Other specialist doctor	Blood culture;<br>Cephalotin, 100 ml vial;<br>Chest X-ray;<br>Gentamycin, injection, 40 mg/ml in 2 ml vial;<br>IV giving/infusion set, with needle;<br>Oxygen, 1000 liters, primarily with oxygen cylinders
31	Case management of severe neonatal infection (sepsis/pneumonia) with full supportive care	MNCH _31. CMSNI	Case management of neonates with suspected sepsis/pneumonia treated with hospital-based full supportive care, including oxygen, IV fluids, IV antibiotics, blood transfusion, phototherapy, etc. as needed, in addition to KMC.	Newborn	Maternal and newborn	Hospital	10 inpatient bed days	160 minutes N/M	Blood culture;<br>Cephalotin, 100 ml vial;<br>Chest X-ray;<br>Gentamycin, injection, 40 mg/ml in 2 ml vial;<br>IV giving/infusion set, with needle;<br>Oxygen, 1000 liters, primarily with oxygen cylinders
32	Facility based management of neonatal infection (sepsis/pneumonia) with injectible (and oral) antibiotics	MNCH _32. CMNI	Treatment of sepsis and infection at first level facility, with two days injectable antibiotics followed by oral amoxicillin for 7 days.	Newborn	Child	Primary level care	3 outpatient visits	-	Gentamycin, injection, 40 mg/ml in 2 ml vial;<br>Procaine benzylpenicillin, powder for injection, 1 g (= 1 million IU) in vial
33	Management of diarrhea through Oral Rehydration Solution and Zinc	MNCH _33. ORSzinc	Management of mild and moderate diarrhea with oral rehydration solution (ORS) and zinc tablets	Children 0-59 months	Child	Community level	1 outpatient visit	-	ORS, sachet; Zinc, tablet, 20 mg

34	Community based management of pneumonia	MNCH _34. CCM_P	Home visits for diagnosis and treatment of community based management of pneumonia in children below the age of five years, provided by community health workers	Children 0-59 months	Child	Community level	2 outpatient visits	-	Amoxicillin, caplet, 250 mg;<br>Paracetamol, tablet, 100 mg;<br>Salbutamol, tablet, 4 mg;<br>Salbutamol, syrup, 2 mg/5 ml
35	Antibiotics for treatment of dysentery	MNCH _35. DYS	Children with diarrhea presenting with blood in the stool receive a three day course of ciprofloxacin and are re-evaluated after 2 days.	Children 0-59 months	Child	Primary level care	2 outpatient visits	-	Ciprofloxacin, tablet, 250 mg
36	Facility based management of pneumonia	MNCH _36. FCM_P	Management of pneumonia with oral antibiotics.	Children 0-59 months	Child	Primary level care	2 outpatient visits	-	Amoxicillin, caplet, 250 mg;<br>Paracetamol, tablet, 100 mg;<br>Salbutamol, tablet, 4 mg;<br>Salbutamol, syrup, 2 mg/5 ml
37	Management of children with severe acute malnutrition	MNCH _37. CMSAM	Integrated management of children with Severe Acute Malnutrition (<-3 Z-score) through outpatient care for cases without medical complication (80%), and inpatient care for cases with medical complications and/or infants younger than 6 months (20%).	Children 0-59 months	Nutrition; Child	Primary level care, incl. community	8 outpatient visits + 2.8 inpatient bed days	480 minutes N/M ; 360 minutes Other specialist doctor	Therapeutic spread, sachet 92g/CAR-150<br>Amoxicillin, caplet, 250 mg<br>Mebendazole, chewable tablet, 500 mg<br>F-100 therapeutic diet, sach., 114g/CAR-90<br>F-75 therapeutic milk, 102.5 g<br>Nystatin 100,000 iu/ml<br>Fluconazole 2 mg/ml<br>Amoxicillin, powder/oral suspension, 125 mg/5 ml<br>Benzathine benzylpenicillin, powder for injection, 2.4 million IU<br>Sulfamethoxazole + trimethropin, tablet, 100 mg + 20 mg<br>Gentamycin, injection, 40 mg/ml in 2 ml vial<br>Ampicillin, powder for injection, 500 mg, vial<br>Metronidazole, injection, 500 mg in 100 ml vial<br>Metronidazole, oral solution 200 mg/5 ml

									Ciprofloxacin, tablet, 250 mg<br>Artusenate, 20 mg/ml, 1 ml ampoule<br>Glucose inj 5 %, 500 ml with giving set<br>CareStartMal Pf (HRP2/pf-pLDH), kit/60<br>Tetracycline eye ointment, 1 %, tube 5 mg<br>Gentamicine eye drops 0.3%/5ml<br>Zinc oxide ointment, 10%<br>Miconazolenitrate cream 2%/TBE-30g<br>Folic acid, tablet, 5 mg<br>Vitamin A, caplet, 100,000 IU<br>Vitamin A, caplet, 200,000 IU<br>Sodium lactate injection (Ringer's), 500 ml, with giving set<br>Furosemide inj 10mg/ml 2ml amp<br>Paraffin compress<br>Cloxacillin<br>ReSoMal, 42 g sachet for 1 liter<br>Erythromicine solution 125 mg/ml<br>Ceftriaxone, powder for injection, 250 ml vial<br>Magnesium sulfate, injection, 500 mg/ml in 10-ml ampoule<br>Permetrine cream 5%<br>Permethrin shampoo/sol. 1%, 100ml bottle<br>Ciprofloxacin, 250 mg / 5 ml<br>Albendazole, tablet, 400 mg<br>prothionamide 125 mg tablet<br>Artemether + Lumefantrine, tablets, 20 + 120 mg, 6 x 1 blister
	**Packages**							-	

**P1**	Preventing and managing unplanned pregnancy	MNCH _P1. UPP	Family planning counseling integrated into safe abortion and post-abortion care (3 interventions)	Women seeking to plan pregnancy	Maternal and newborn	Primary level care; (Hospital level for post-abortion care)	country-specific depends on methods mix	-	As listed above for individual interventions
**P2**	Comprehensive antenatal care	MNCH _P2. ANC	A package of antenatal care aligned with WHO guidelines and including tetanus toxoid vaccine, iron supplementation, calcium supplementation,balanced energy supplementation, syphilis detection and treatment, hypertensive disorder case mgt, MgSO4 mgt of pre-eclampsia, and IPT where relevant (8 interventions)	Pregnant women	Maternal and newborn	Primary level care	8 outpatient visits	-	As listed above for individual interventions
**P3**	Skilled assistance for normal delivery	MNCH _P3. SBA	Skilled assistance with facility based births, not neccessarily EmOC level. Components include immediate assessment and stimulation, support during labor and delivery, active management of the third stage of labour, newborn resuscitation, and clean cord care. (5 interventions)	Pregnant women	Maternal and newborn	Primary level care	1 inpatient bed day	Normal delivery includes 360 minutes M/N and 30 minutes MD	As listed above for individual interventions
**P4**	Skilled assistance for normal delivery + family planning	MNCH _P4. SBA + FP	P3+ integrated postpartum family planning advice and contraceptive provision (6 interventions)	Pregnant women	Maternal and newborn	Primary level care	country-specific depends on methods mix	-	As listed above for individual interventions
**P5**	Skilled delivery + management of complications	MNCH _P5. SBA + comp	Skilled assistance for normal deliveries with quick and efficient referral to quality emergency obstetric care services when complications arise, + induction of labor + full	Pregnant women	Maternal and newborn	Primary level care + Hospital	Country-specific, (depends on prevalence of complications)	Induction of labor includes 40 minutes M/N and 20	As listed above for individual interventions

			supportive care for newborn infections (12 interventions)					minutes MD	
**P6**	Skilled delivery + management of complications + family planning	MNCH _P6. SBA + comp + FP	P5+ integrated postpartum family planning advice and contraceptive provision (13 interventions).	Pregnant women	Maternal and newborn	Primary level care + Hospital	country-specific depends on methods mix	-	As listed above for individual interventions
**P7**	Case management of newborn complications at referral level	MNCH _P7. CMNC	Full supportive care for premature babies + Case management of severe neonatal infection (sepsis/pneumonia) with full supportive care (2 interventions).	Newborns with complications (prematurity, severe infection)	Maternal and newborn	Hospital	6 inpatient bed days	-	As listed above for individual interventions
**P8**	Community based newborn and child care	MNCH _P8. CBNCC	Community based preventive and curative care (breastfeeding promotion, postnatal visits, vitamin A supplementation, management of infections, pneumonia and diarhea), (5 interventions).	Newborns and children 0-59 months	Maternal and newborn; Child	Community	Country-specific	-	As listed above for individual interventions
**P9**	Infant and young child feeding	MNCH _P9. IYCF	Breastfeeding promotion + Complementary feeding promotion + Vitamin A supplementation (3 interventions).	Newborns and children 0-59 months	Nutrition; Child	Community and primary level care	9 outpatient visits	-	As listed above for individual interventions
**P10**	Routine EPI (measles, diphteria, pertussis, tetanus, and tuberculosis)	MNCH _P10. EPI	BCG, DTP, Hib, and Measles immunization (4 interventions).	Newborns and children 0-59 months	Immunization	Primary level care	6 outpatient visits	-	As listed above for individual interventions
**P11**	Routine EPI + additional vaccines	MNCH _P11. EPI+ROTA+PCV	BCG, DTP, Hib, Measles, Rotavirus and Pneumonococcal vaccines (6 interventions).	Newborns and children 0-59 months	Immunization	Primary level care	6 outpatient visits	-	As listed above for individual interventions

**P12**	Primary level integrated management of the sick child (includes link to the community)	MNCH _P12. IMCI	Management of diarrhea, dysentery, pneumonia, and severe malnutrition (4 interventions).	Children 0-59 months	Child	Primary level care	Country-specific	-	As listed above for individual interventions

* Notes on Specialized Staff time: MD = Medical Doctor; N/M = Nurse/Midwife; the category of "Other staff" includes Assistant nurses and midwives, Laboratory technicians and assistants

Table S2.2. Prices used in analysis (2010 I$)

Country	Outpatient visit (health centre, no beds)*	Inpatient bed day (primary level hospital) *	Annual wage MD**	Annual wage N/M**	Annual wage Other staff**
Cambodia	3.7	19.5	7,010	5,695	3,269
Indonesia	6.7	44.5	5,341	4,339	2,490
Laos	4.2	23.2	7,232	5,875	3,372
Malaysia	20.2	201.7	27,289	22,168	12,724
Maldives	11.7	95.3	14,180	11,519	6,612
Myanmar	2.2	9.6	2,655	2,157	1,238
Philippines	6.2	40.2	11,367	9,234	5,300
Sri Lanka	7.8	54.3	5,380	4,370	2,508
Thailand	12.6	105.4	8,005	6,503	3,732
Timor-Leste	2.7	12.9	4,168	3,386	1,943
Viet Nam	5.1	30.3	8,443	6,859	3,937

Burundi	1.1	3.7	2,627	2,134	1,225
Comoros	2.2	9.5	5,375	4,367	2,506
Djibouti	4.0	21.7	12,775	10,378	5,956
Eritrea	1.0	3.4	3,119	2,534	1,454
Ethiopia	1.9	7.7	3,324	2,700	1,550
Kenya	2.9	14.0	5,475	4,448	2,553
Madagascar	1.8	7.2	3,547	2,881	1,654
Malawi	1.7	6.5	3,418	2,777	1,594
Mauritius	19.5	192.3	22,172	18,011	10,338
Mozambique	1.7	6.8	4,338	3,524	2,023
Rwanda	2.2	9.5	4,134	3,358	1,927
Seychelles	28.7	328.6	34,897	28,348	16,271
Somalia	1.9	7.7	3,324	2,700	1,550
South Sudan	4.2	23.5	26,708	21,696	12,453
Sudan	4.2	23.5	26,708	21,696	12,453
Uganda	2.3	10.3	4,400	3,574	2,051
Tanzania	2.5	11.6	4,653	3,780	2,170
Zambia	2.7	12.9	6,360	5,167	2,965

*Source: Stenberg K, Lauer JA, Gkountouras G, Fitzpatrick C and Stanciole A. Econometric estimation of WHO-CHOICE country-specific costs for inpatient and outpatient health service delivery Cost Effectiveness and Resource Allocation. 2018 16:11

**Source: Serje J, Bertram MY, Brindley C and Lauer JA. Global health worker salary estimates: an econometric analysis of global earnings data. Cost Effectiveness and Resource Allocation 2018 16:10.
